# BERT-AmPEP60: A BERT-Based Transfer Learning Approach to Predict the Minimum Inhibitory Concentrations of Antimicrobial Peptides for *Escherichia coli* and *Staphylococcus aureus*

**DOI:** 10.1021/acs.jcim.4c01749

**Published:** 2025-03-14

**Authors:** Jianxiu Cai, Jielu Yan, Chonwai Un, Yapeng Wang, François-Xavier Campbell-Valois, Shirley W. I. Siu

**Affiliations:** † Faculty of Applied Sciences, Macao Polytechnic University, Rua de Luís Gonzaga Gomes, Macau SAR 99078, China; ‡ Institute of Science and Environment, University of Saint Joseph, Rua de Luís Gonzaga Gomes, Macau SAR 99078, China; § School of Computer Science, Chongqing University, Shapingba, Chongqing 400044, China; ∥ T-Rex Technology HK Limited, Unit 1017-1, 10/F, Building 19W, Hongkong Science Park, Shatin, Hong Kong, New Territories; ⊥ Host-Microbe Interactions Laboratory, Center for Chemical and Synthetic Biology, Department of Chemistry and Biomolecular Sciences, University of Ottawa, Ottawa, Ontario K1N 6N5, Canada; # Centre for Infection, Immunity, and Inflammation, University of Ottawa, Ottawa K1N 6N5, Ontario, Canada; ∇ Department of Biochemistry, Microbiology and Immunology, University of Ottawa, Ottawa K1N 6N5, Ontario, Canada; ○ Centre for Artificial Intelligence Driven Drug Discovery, Faculty of Applied Sciences, Macao Polytechnic University, Rua de Luís Gonzaga Gomes, Macau SAR 99078, China

## Abstract

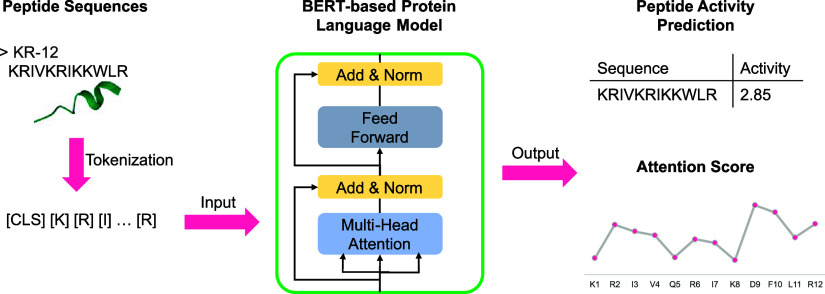

Antimicrobial peptides (AMPs) are a promising alternative for combating bacterial drug resistance. While current computer prediction models excel at binary classification of AMPs based on sequences, there is a lack of regression methods to accurately quantify AMP activity against specific bacteria, making the identification of highly potent AMPs a challenge. Here, we present a deep learning method, BERT-AmPEP60, based on the fine-tuned Bidirectional Encoder Representations from Transformers (BERT) architecture to extract embedding features from input sequences. Using the transfer learning strategy, we built regression models to predict the minimum inhibitory concentration (MIC) of peptides for *Escherichia coli* (EC) and *Staphylococcus aureus* (SA). In five independent experiments with 10% leave-out sequences as the test sets, the optimal EC and SA models outperformed the state-of-the-art regression method and traditional machine learning methods, achieving an average mean squared error of 0.2664 and 0.3032 (log μM), respectively. They also showed a Pearson correlation coefficient of 0.7955 and 0.7530, and a Kendall correlation coefficient of 0.5797 and 0.5222, respectively. Our models outperformed existing deep learning and machine learning methods that rely on conventional sequence features. This work underscores the effectiveness of utilizing BERT with transfer learning for training quantitative AMP prediction models specific for different bacterial species. The web server of BERT-AmPEP60 can be found at https://app.cbbio.online/ampep/home. To facilitate development, the program source codes are available at https://github.com/janecai0714/AMP_regression_EC_SA.

## 1 Introduction

The widespread use of antibiotics has led to the emergence of antimicrobial-resistant (AMR) bacteria, also known as *superbugs*. These bacteria have developed mechanisms to withstand the effects of antibiotics, rendering many previous treatments ineffective.^1^ A recent systematic review of global studies on AMR, utilizing a predictive statistical model, estimated that bacterial AMR was responsible for approximately 4.95 million deaths in 2019.^2^ Without intervention, this number could rise to 10 million by 2050.^3^ Consequently, the development of a new and effective class of antibiotics is urgently needed. Antimicrobial peptides (AMPs) are considered as one of the most promising alternatives to existing antibiotics.^4,5^ AMPs are polypeptides that play a crucial role in the innate immune defense of various organisms, including humans, animals, and plants. Compared to other small molecules, AMPs offer several key advantages as potential therapeutics. One key advantage is their diverse mechanisms of action. The most common mechanism is the disruption of the bacterial membrane, which cause the loss of the membrane potential to kill bacterial cell.^6^ Since the properties of the membrane are determined by several genes, bacteria cannot easily develop resistance mechanisms against this class of AMPs. Additionally, a subset of AMPs targets a variety of intracellular components, such as nucleic acids, protein synthesis, and enzymes.^7^ Moreover, AMPs have a broad spectrum of antimicrobial activity against bacteria, viruses, and fungi. They often show high selectivity for microbial cells over host cells, which reduces the likelihood of side effects.^8^ Therefore, the discovery and development of novel AMPs with high potency and specificity are crucial for advancing new antibiotic treatments.

The recent success of artificial intelligence and the availability of public AMP databases have enabled the development of more accurate predictive methods for discovering new AMPs. These methods can be broadly divided into traditional machine learning-based (ML) and deep learning-based (DL) methods. Traditional ML methods, such as support vector machine (SVM),^9−13^ discriminant analysis (DA),^14^ random forest (RF),^15−18^ K-nearest neighbors (KNN),^12,19,13^ and ensemble learning^20−22^ have been utilized to build classifiers. These methods necessitate prior domain knowledge for manually selecting input features before training the models. In contrast, DL-based methods leverage the capability of neural networks to automatically learn intricate patterns and capture complex features directly from sequences.^23^ Numerous DL-based methods employing different architectures, such as convolutional neural networks (CNN),^24−27^ recurrent neural networks (RNN),^28^ and hybrid architectures,^29,30^ have been proposed for AMP prediction. More recently, protein language models (PLMs), inspired by advancements in natural language processing (NLP), have garnered significant attention. Prominent examples include TAPE,^31^ ProtTrans,^32^ and ESM2.^33^ These models leverage large data sets of protein sequences to extract meaningful representations that can capture evolutionary, structural, and functional information. Consequently, they have demonstrated the ability to learn complex relationships within protein sequences and have proven successful in various downstream prediction tasks, including protein structure and function prediction.^34,35^

Most of these prediction methods focus on the classification of peptides. To our knowledge, only a few studies have proposed the quantitative prediction of antimicrobial peptide activity, such as the minimum inhibitory concentration (MIC). In 2017, Nagarajan et al.^36^ developed a Bi-LSTM regression model for predicting the activity of AMP against *Escherichia coli* (EC). They used a data set of 501 sequences with a maximum sequence length of 30 amino acids. The performance of the model was not reported in the paper; however, it was used to rank and select sequences for experimental validation. Out of the ten selected peptides, two were confirmed to exhibit good potency against EC and showed broad-spectrum antimicrobial activity against a range of multidrug resistant isolates. Later in 2021, Dean et al.^37^ developed eight different regression models to predict the MIC values of AMPs: CNN, Elastic Net (ENet), Gradient Boosting (GB), Kernel Ridge (KR), Lasso, RF, Light Gradient Boosting Machine (LGBM), and Extreme Gradient Boosting (XGB). They compiled a data set of 3280 sequences with a maximum of 40 residues in length against EC. Among the models, GB achieved both the highest mean *R*^2^ value of 0.73 and the lowest root mean squared error (RMSE) value of 0.50 (log μM). Recently, we developed a Multi-Branch CNN-Attention (MBC-Attention) method to regress the MIC values of AMPs against EC using an ensemble of CNNs across multiple input branches integrated with attention modules.^38^ The optimal model achieved an average Pearson correlation coefficient (PCC) of 0.775 and a RMSE of 0.533 (log μM).

Despite the progress made in AMP research, current regression methods for predicting AMP activity are limited in their accuracy and are not yet suitable for the de novo design of novel AMPs. Therefore, the development of more accurate methods is warranted. However, the major hurdle in advancing ML methods lies in the scarcity of experimental antimicrobial data. In this study, we aim to tackle the challenge of data scarcity through a transfer learning strategy. Transfer learning involves adopting a pretrained model, previously trained to learn sequence representations from large protein sequence databases, to encode sequences from smaller data sets such as AMPs. ProtBERT^32^ is a large language model for protein sequences using bidirectional encoder representation of transformers (BERT) architecture.^39^ It was trained extensively on a vast data set comprising millions of protein sequences from UniProt. Here, we fine-tuned ProtBERT to learn AMP sequences for two pathogenic bacteria EC and *Staphylococcus aureus* (SA). These two bacterial models are representative of Gram-negative and Gram-positive bacteria, respectively, which are known key differences in the properties of their membrane, the main target of AMPs. They also show a high incidence of antibiotic resistance in clinical settings, making them ideal comparative models. Additionally, we incorporated a neural network module to regress the extracted sequence patterns to the experimental MIC values of the sequences. Moreover, as BERT’s multihead self-attention mechanism allows the model to learn the relative importance of each token in an input sequence, we exploited the attention weights to reveal the contribution of individual amino acids to the antimicrobial function of peptides in two case studies.

## 2 Materials and Methods

### 2.1 Data Set Preparation

The Database of Antimicrobial Activity and Structure of Peptides (DBAASP)^40^ is a freely accessible and comprehensive database with detailed information on the antimicrobial activities of peptides. A total of 152,486 AMP entries were downloaded from DBAASP and categorized into 704 groups by target species. Of these groups, 8446 and 7642 are data entries of AMP against EC and SA, respectively. The data were preprocessed using the following procedures. First, all entries with one of the following characteristics were excluded from the data set: presence of unnatural amino acids (‘B’, ‘J’, ‘O’, ‘U’, ‘Z’, ‘X’), sequence length >60 aa or <5 aa entries, or experimental MIC values against EC or SA that were missing or >10,000 μM. The activity values of the remaining peptides were converted to pMIC using eq 1, where the unit of MIC is μM. For a sequence that has multiple MIC values due to different reference sources or different subtypes of the same target species, these values were averaged to obtain the mean activity measure of the sequence.
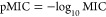
1

After preprocessing, 4042 AMPs against EC and 3275 AMPs against SA were obtained. The length of these peptides is between 5 and 60 aa. To train and test predictive models, the preprocessed data sets were randomly divided into train and test sets. The test set consists of 10% of the entire data set, while the remaining set after subtracting the test data is used as the train set. Table 1 summarizes the final data sets used in this study.

**Table 1:** Summary of the AMP Data Sets for *E. coli* and *S. aureus*

name	description	EC	SA
all subtypes	downloaded from DBAASP in July 2023	9746	8838
whole	after preprocessing	4042	3275
test	10% randomly selected from the whole set	404	328
train	data not included in the test sets	3638	2947

**Figure 1:**
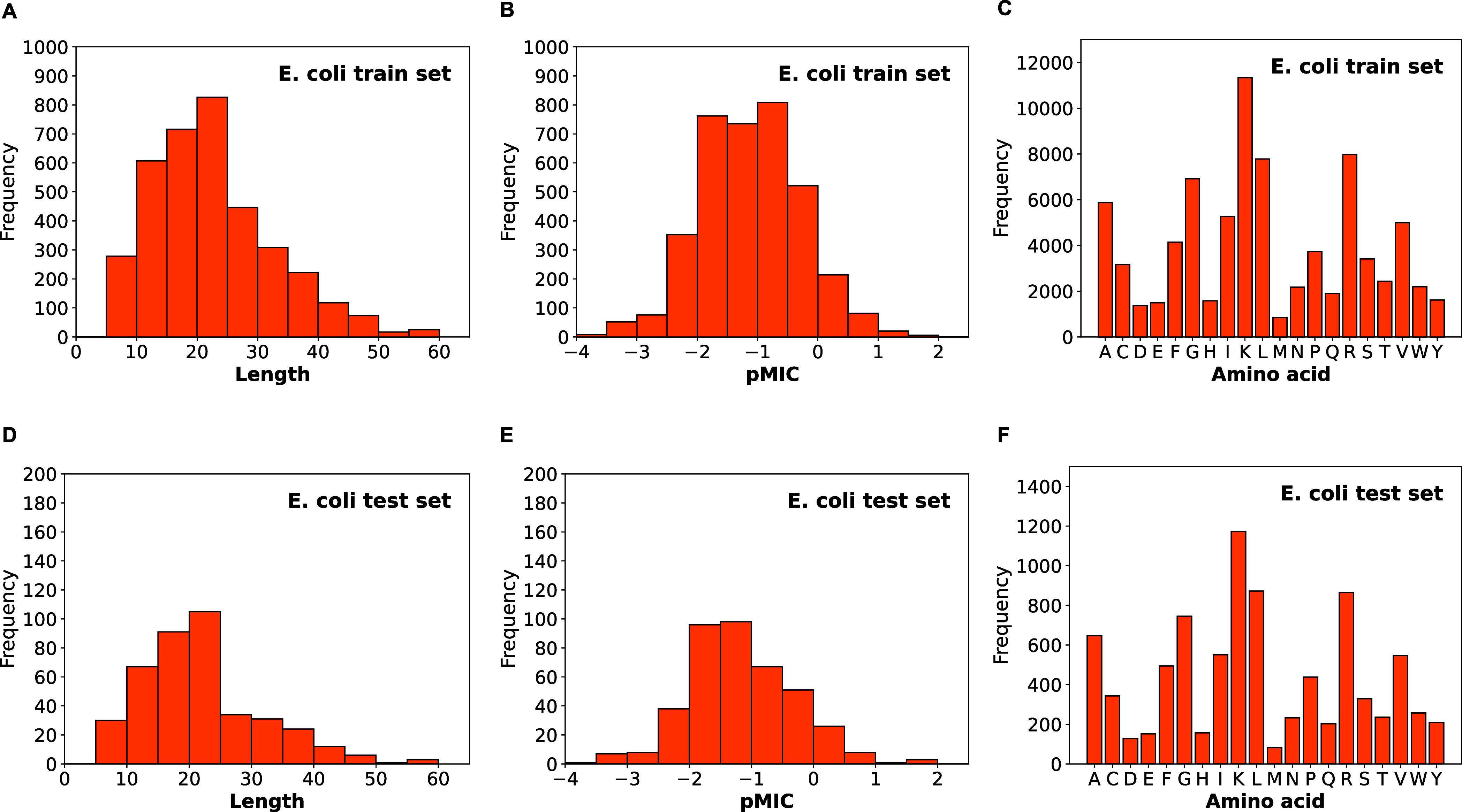
Distributions of AMPs for *E. coli* as a function of sequence length, pMIC, and amino acid composition in the train set (A–C) and test set (D–F). One of the five random data partition results is displayed.

**Figure 2:**
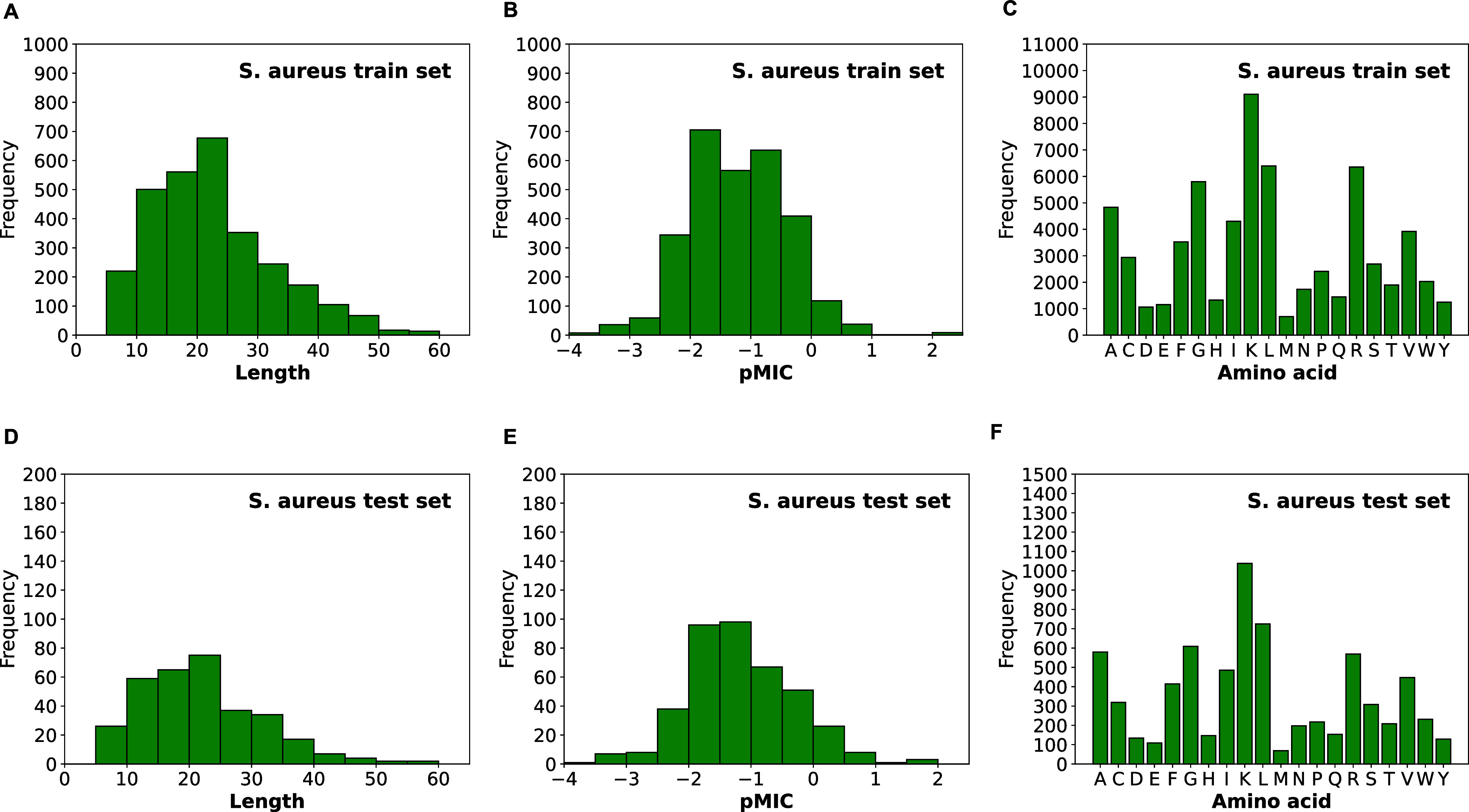
Distributions of AMPs for *S. aureus* as a function of sequence length, pMIC, and amino acid composition in the train set (A–C) and test set (D–F). One of the five random data partition results is displayed.

We examined the data distributions after applying the random splitting strategy. As shown in Figures 1 and 2, the train and test sets maintain the same distributions with respect to sequence length, pMIC, and amino acid composition. Most sequences in the train set and the test set exhibit low sequence similarity, with an average sequence identity of only 17% for both EC and SA data sets. The distribution of sequence identity between sequences in the train set and the test set can be found in Figure S1. Overall, the AMP sequences for EC have a median length of 22 residues and a median pMIC value of −1.13 (corresponding to MIC of 13.49 μM), while the AMP sequences for SA have a median length of 22 residues and a median pMIC value of −1.21 (MIC of 16.22 μM).

Finally, to ensure accurate statistical analysis, the data splitting procedure was repeated five times to generate five sets of train and test data. The experiment was conducted independently on each of the five replicates, and the average performance values were then reported.

### 2.2 BERT-Based Transfer Learning Approach for AMP Activity Prediction

**Figure 3:**
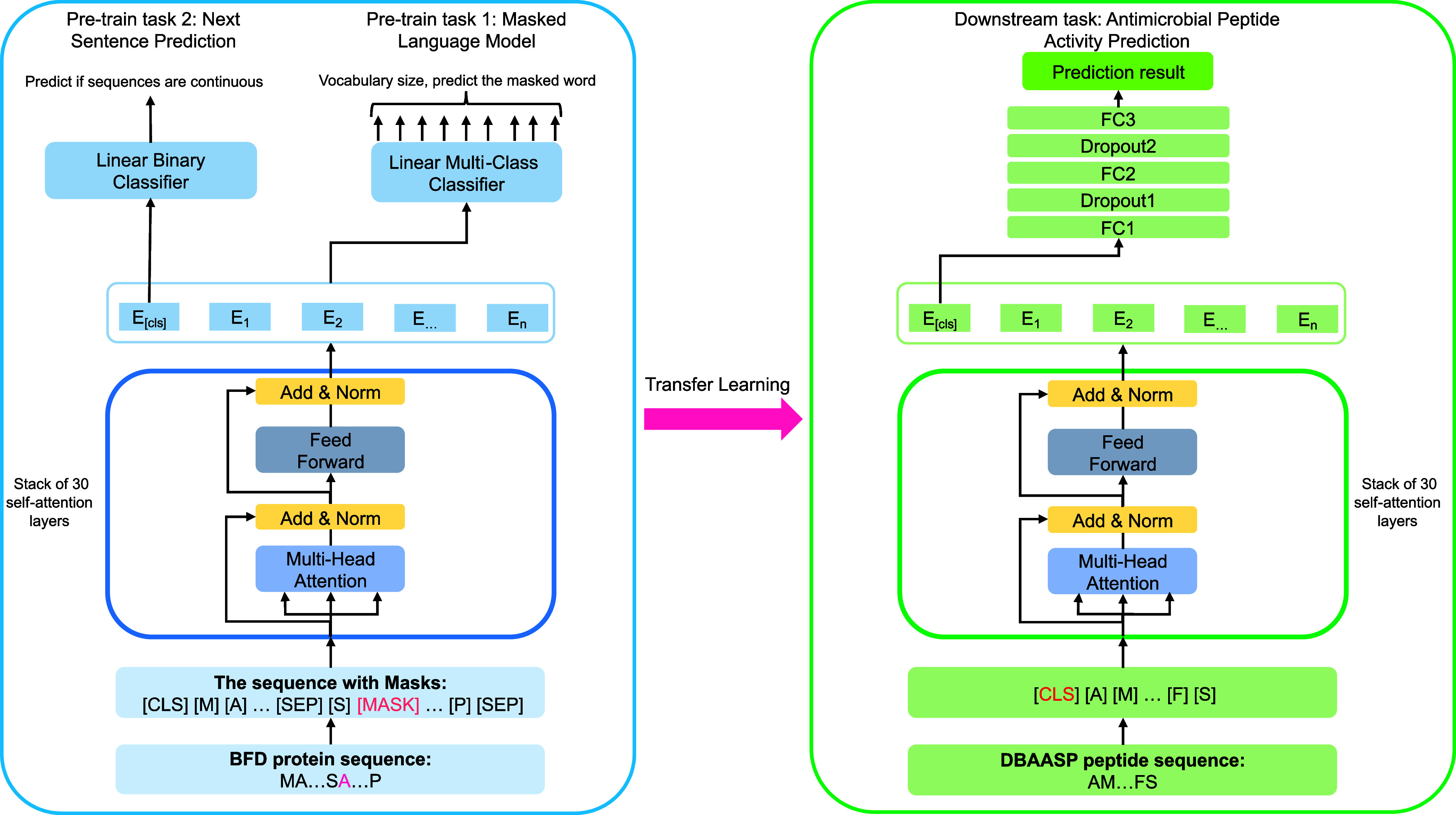
Overview of the transfer learning workflow for training the BERT-AmPEP60 models. Each amino acid sequence is first tokenized for data representation, and the BERT encoder layers derived from the pretrained ProtBERT model are fine-tuned for the downstream AMP regression task.

BERT is the first bidirectional model in natural language processing (NLP) that attempted to reconstruct corrupted tokens as a pretraining task.^39^ In the pretraining process, a sample input sentence is represented as a sequence of tokens, with a special class token (CLS) marking the beginning of the sentence and some tokens randomly masked, denoted as *X* = [*CLS*, *x*_1_, *x*_2_, *x*_*masked*_,..., *x*_*n*_]. Here, *n* represents the total number of tokens in the input sequence. BERT employs word embedding and position embedding to convert each token into a vector representation, incorporating positional information on tokens in the input sequence to the embedding output. Subsequently, the embedding output undergoes a stack of encoder layers, each containing a multiheaded self-attention sublayer, a feed-forward network, a layer normalization, and residual connections (as illustrated in Figure 3). Each encoder layer *l* produces a sequence of contextualized embedding vectors . These contextualized embedding vectors are then used for two primary tasks: masked language model (MLM) and next sentence prediction (NSP). In the MLM task, the masked tokens are predicted by maximizing the likelihood function *p* = *p*(*x*_*masked*_|*x*_*unmasked*_). By omitting some words in a sentence, it is forced to learn and understand the contextual relationships between words in a sentence. On the other hand, in the NSP task, the input sample consists of two sentences separated by a [SEP] token. The objective is to determine whether the second sentence follows the first sentence in the original text or if it is a randomly chosen sentence from the corpus. The model’s output is the embedding vector of the [CLS] token, which is used to make this decision. The NSP task trains the model’s ability to understand relationships between pairs of sentences.

BERT employs a stacked-encoder framework where each encoder layer utilizes multihead self-attention.^41^ Given an input sequence *X* = [*x*_1_, *x*_2_, ···, *x*_*n*_], the attention mechanism assigns an attention weight α_*i*,*j*_ to each token pair *i* and *j*, the α_*i*,*j*_ indicates the relevance of token *i* and *j* and ∑_*j*_ α_*i*,*j*_ = 1. These weights α_*i*,*j*_ are calculated from the scaled dot-product of the Query vector (*Q*) of *i* and the Key vector (*K*) of *j*. A softmax operation is followed to normalize the attention weights so they are all positive and added up to 1 (refer to eq 2). Finally, the attention weights are used to compute a weighted sum of the value vectors (*V*) of *j*. This sum represents the attention output for the current token *i* (refer to eq 3).
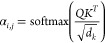
2
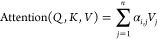
3where *d*_*k*_ is the dimension of *K*, and the resulting vector is passed to the feed-forward networks. For instance, ProtBERT is composed of 30 encoder layers, each with 16 attention heads. Each head learns a unique set of weights, resulting in a total of 480 (30 × 16) distinct sets of attention weights.

To date, only a limited amount of labeled data is available for quantitative prediction of AMP activity. With the advancement of sequencing techniques, the amount of accessible protein sequences, such as those in UniProt, has increased tremendously. These large protein sequence data sets can be used to build and further train protein language models in a self-supervised manner to perform domain-specific tasks such as protein function predictions.^42^ To build a BERT-based protein language model, each sample sequence is treated as a sentence with a special [CLS] token at the start position, and each amino acid is treated as a word token. The [CLS] token represents the aggregated feature of all amino acids in the input sequence.^43^ Here, the [CLS] token feature was used for the downstream AMP activity regression task. We set the maximum length of an input sequence to 60 aa. Sequences less than 60 aa were padded with [PAD] tokens to ensure uniform feature dimensions across all sequences. All tokenized sequences with 60 tokens were feed into the model. Finally, the last hidden state of the [CLS] token was used for downstream tasks.

#### 2.2.1 Fine-Tuning for the AMP Activity Prediction Task

Although the protein sequence language shares similarities with human language, there are distinct differences that make it impractical to directly adopt a pretrained human language model for peptide-related downstream tasks. It is crucial that the knowledge domain of the pretrained model aligns with the domain of the downstream task, in this case is the quantitative prediction of AMP activity. To this end, we employed the ProtBERT model from ProtTrans,^32^ a BERT-based model pretrained on approximately 2.17 billion protein sequences from UniRef100. A schematic view of the ProtBERT architecture and its pretraining tasks are shown in the left side of Figure 3. This pretrained model captures latent information from the input sequence and the relationships between the residue pairs in different representation subspaces. The model is accessible through the Hugging Face Transformers API.^44^

Taking the ProtBERT model, we performed transfer learning to fine-tune ProtBERT and train the quantitative AMP prediction model downstream as shown in the right side of Figure 3. It is worth-mentioning that as the [CLS] token is an aggregated feature representation of all words in an input sequence, only the embedding feature from the [CLS] token is extracted for supervised learning and subsequent prediction. Our downstream prediction model consists of two fully connected layers (512 dimensions for FC1 and 128 dimensions for FC2), each followed by a dropout layer to avoid the problem of overfitting.^45^ The final prediction is calculated in the last layer FC3 to generate an output.

For model optimization, we used the AdamW algorithm,^46^ and the mean squared error (MSE)^47^ was taken as the loss function:
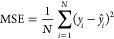
4where *y*_*i*_ represents the ground truth value of sample *i*th,  represents the predicted value of sample *i*th from the regression model, and *N* is the number of samples.

#### 2.2.2 Hyperparameter Tuning

When fitting ProtBERT to our AMP activity regression task, overfitting becomes a significant concern due to the large size of the model, comprising millions of parameters, contrasted with the relatively small data sets for EC and SA, each contains only thousands of peptide entries. To avoid overfitting, two regularization strategies were used: weight decay and dropout. By adding a regularization term to the loss function, weight decay penalizes large weights in the model and thus prevents overcomplexity of the model to achieve good generalization performance.^48^ On the other hand, dropout involves randomly deactivating or ”dropping out” some neurons in a neural network during training iterations. This prevents coadaptation between neurons leading to improve robustness of the model.^49^

To identify the best model architecture, we first assessed the model’s performance with 1, 2, 3, and 4 FC layers using the default parameters. Subsequently, after determining the optimal number of layers, grid search was performed on the learning rate, weight decay rate, dropout rate, and loss function. Each parameter was given a range of candidate values, as detailed in Table 2. The train set (Table 1) was further split in a ratio of 9:1 for training and validation purposes. Therefore, the EC model was trained on 3274 sequences and validated on 364 sequences, while the SA model was trained on 2653 sequences and validated on 294 sequences. Each train-validate experiment was repeated 5 times. A maximum epoch of 100 iterations was used in training.

**Table 2:** Hyperparameters and Candidate Value Ranges Considered During Model Optimization

hyperparameter	candidate value	optimal value
number of FC layers	1, 2, 3, 4	3
weight decay rate	0.1, 0.3, 0.5, 0.7, 0.01, 0.03,... 1e–3, 3e–3,...,7e–3	3e–3
dropout rate	0.1, 0.2, 0.3, ..., 0.9	0.2
learning rate	1e–2, 1e–3, 1e–4, 1e–5, 1e–6	1e–5
loss function	MSE, MLSE, CCC	MSE

In total, there were 8100 experiments (12 weight decay rates ×9 dropout rates ×5 learning rates ×3 loss functions ×5 repeats) to optimize the parameters. After exhaustive experiments, the optimal hyperparameters were determined to be 3 FC layers, a learning rate of 1e–5, a dropout rate of 0.2, and a weight decay rate of 3e–3, with a batch size of 64 resulting in the best prediction performance in terms of MSE.

After all hyperparameters were determined, the final model was trained using the whole train set with the early stopping criteria. The generalization performance for the optimal EC and SA models was evaluated using 404 and 328 test sequences, respectively. A learning curve of our models is shown in Figure S2. It illustrates the performance of our models across different epochs. As depicted, our models converged quickly, and no overfitting was observed.

### 2.3 Model Performance Metrics

We used MSE, Pearson Correlation Coefficient (PCC),^50^ and Kendall’s Correlation Coefficient (KTC)^51^ as metrics to measure the performance of the regression models.
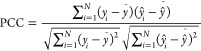
5where *y*_*i*_ represents ground truth value of sample *i*th;  denotes the *i*th sample of prediction values of regression model, *n* is the number of samples.
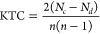
6where *N*_*c*_ is the number of concordant pairs, *N*_*d*_ the number of discordant pairs, *n* is the sample number.

## 3 Results

### 3.1 Comparative Analysis of Prediction Models

To determine the baseline performance, we first conducted experiments on the curated data sets using traditional ML algorithms and common peptide descriptors. For ML algorithms, we selected DT (Decision Tree), SVM, RF, and KNN. For peptide descriptors, we used QSOrder (Quasi-sequence-order descriptors) and the CTD descriptors, i.e., composition (CTDC), transition (CTDT), and distribution (CTDD). These descriptors are among the most important features in the classification of AMP^15^ and the quantitative prediction of anticancer peptides.^52^ For each combination of ML algorithm and descriptor, the hyperparameters of the model were selected by grid search. The train-validate-test experiment was repeated five times. The mean and standard deviation of the best performing DT, SVM, RF, and KNN models for EC and SA are presented in Table 3. The MSE values of these traditional ML models for EC are between 0.3439 and 0.6888, and those for SA between 0.3747 and 0.7493. It is interesting that the best algorithm-descriptor combination turns out to be RF and CTDD for both EC and SA. This is the same combination that we found in our previous AMP study for the classification model.^15^ Meanwhile, the DT models performed worst in both regression tasks for EC and SA.

**Table 3:** Comparative Analysis of Machine Learning, Deep Learning, and Protein Language Models for Predicting MIC of Peptides across Five Independent Test Experiments

model	feature^a^	MSE	std^b^	PCC	std	KTC	std
*E. coli*							
DT	QSOrder	0.6888	0.0479	0.5422	0.0340	0.3501	0.0257
SVM	QSOrder	0.3912	0.0141	0.6831	0.0285	0.4620	0.0198
RF	CTDD	0.3439	0.0237	0.7352	0.0076	0.5131	0.0139
KNN	QSOrder	0.4186	0.0500	0.6698	0.0208	0.4409	0.0228
MLP	ESM2	0.3764	0.0196	0.7027	0.0197	0.4848	0.0194
MBC	Feature combination^c^	0.3514	0.0184	0.7430	0.0199	0.5373	0.0241
BERT-AmPEP60	ProtBERT	**0.2664**	0.0141	**0.7955**	0.0121	**0.5797**	0.0104
*S. aureus*							
DT	CTDD	0.7493	0.0326	0.4476	0.0567	0.2754	0.0473
SVM	QSOrder	0.4801	0.0397	0.5591	0.0349	0.3880	0.0245
RF	CTDD	0.3747	0.0183	0.6879	0.0218	0.4576	0.0346
KNN	QSOrder	0.4652	0.0228	0.5910	0.0219	0.3778	0.0117
MLP	ESM2	0.3924	0.0084	0.7024	0.0129	0.5101	0.0061
MBC	Feature combination	0.3663	0.0389	0.6901	0.0416	0.4817	0.0323
MBC-Attention	Feature combination	0.3546	0.0364	0.7086	0.0379	0.4926	0.0284
BERT-AmPEP60	ProtBERT	**0.3032**	0. 0058	**0.7530**	0.0079	**0.5222**	0.0125

a Feature: Protein sequence descriptors or embeddings from the fine-tuned ProtBERT model.

b std: standard deviation of the experiments with five replicates.

c Feature combination: the best-14 feature types based on the PseKRAAC (Pseudo K-tuple Reduced Amino Acids Composition) and QSOrder.

To further evaluate the discriminative power of the proposed BERT-based transfer learning approach, we conducted the train-validate-test experiment in the same way as for the ML models. The validation performance is presented in Table S1 and the test performance in Table 3. As observed, our proposed model for EC achieved an average MSE of 0.2664, PCC of 0.7955, and KTC of 0.5797 on the test set. In comparison, the model for SA achieved an average MSE of 0.3032, PCC of 0.7530 and KTC of 0.5222 on the test set. It is worth noting that the test performance closely matches the validation performance, implying that our models generalize well and no overfitting occurred.

We also compared our models with existing DL methods and the more recent protein language model ESM2. First, we implemented ESM2 in combination with a multilayer perceptron in the same task-specific fine-tuning setup as our proposed model. Additionally, we selected the MBC and MBC-Attention methods proposed by Yan et al.,^38^ which represent state-of-the-art CNN approaches for AMP regression tasks with publicly available code. The MBC method was originally designed for classifying peptides that bind ion channels, while the MBC-Attention method enhances the MBC model for predicting the MIC of AMPs against EC. We adapted and retrained ESM2, MBC, and MBC-Attention models using our train data set and evaluated their performance on the test set. Interestingly, ESM2 did not outperform the CNN-based MBC models, while the MBC-Attention models for EC and SA demonstrated improvements over their MBC counterparts. Despite these improvements, our proposed BERT-based models remain the best models for predicting AMP activity against EC and SA.

**Figure 4:**
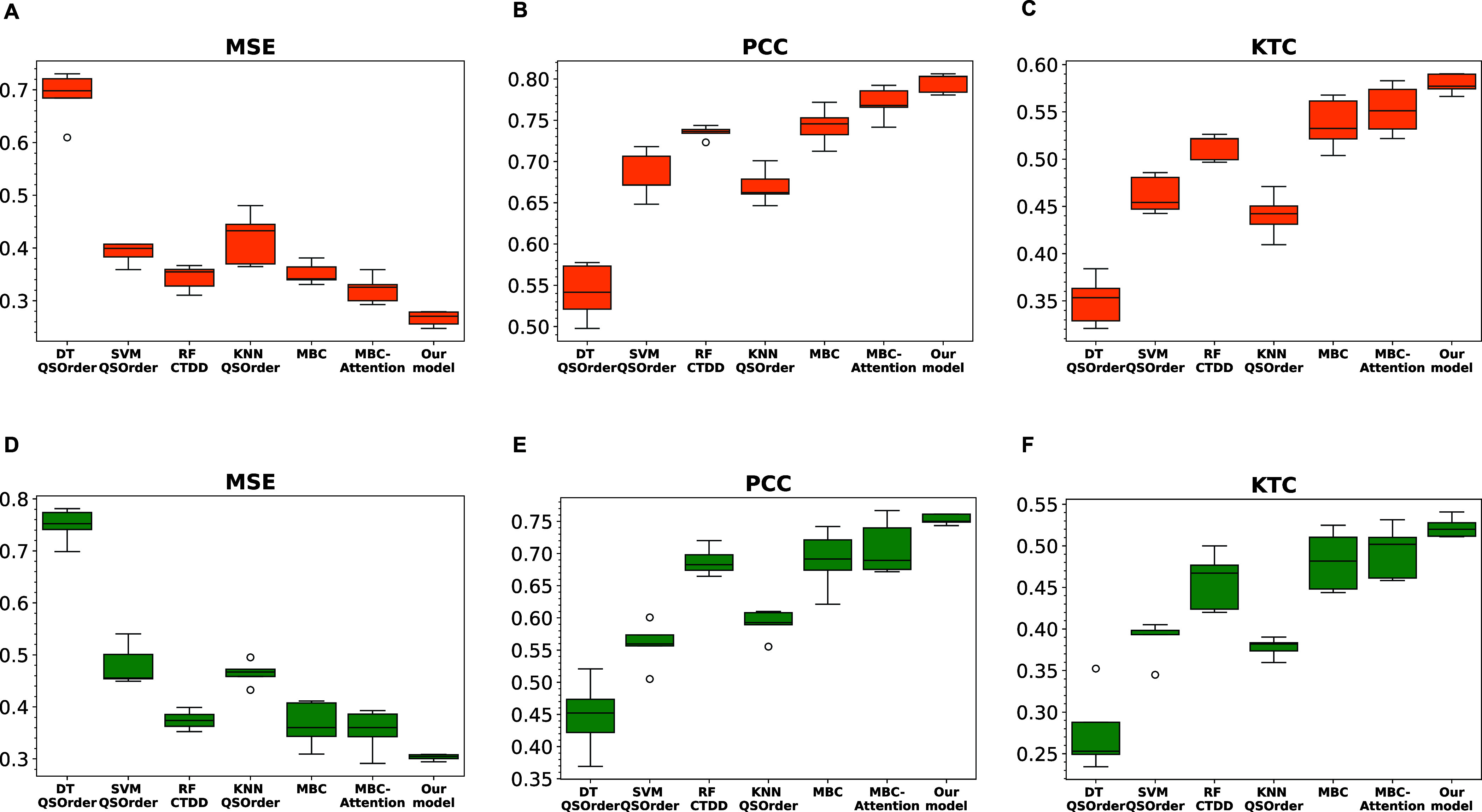
Performance comparison of all predictive models on the test set across five repeated experiments. (A–C) for *E. coli* and (D–F) for *S. aureus*.

**Figure 5:**
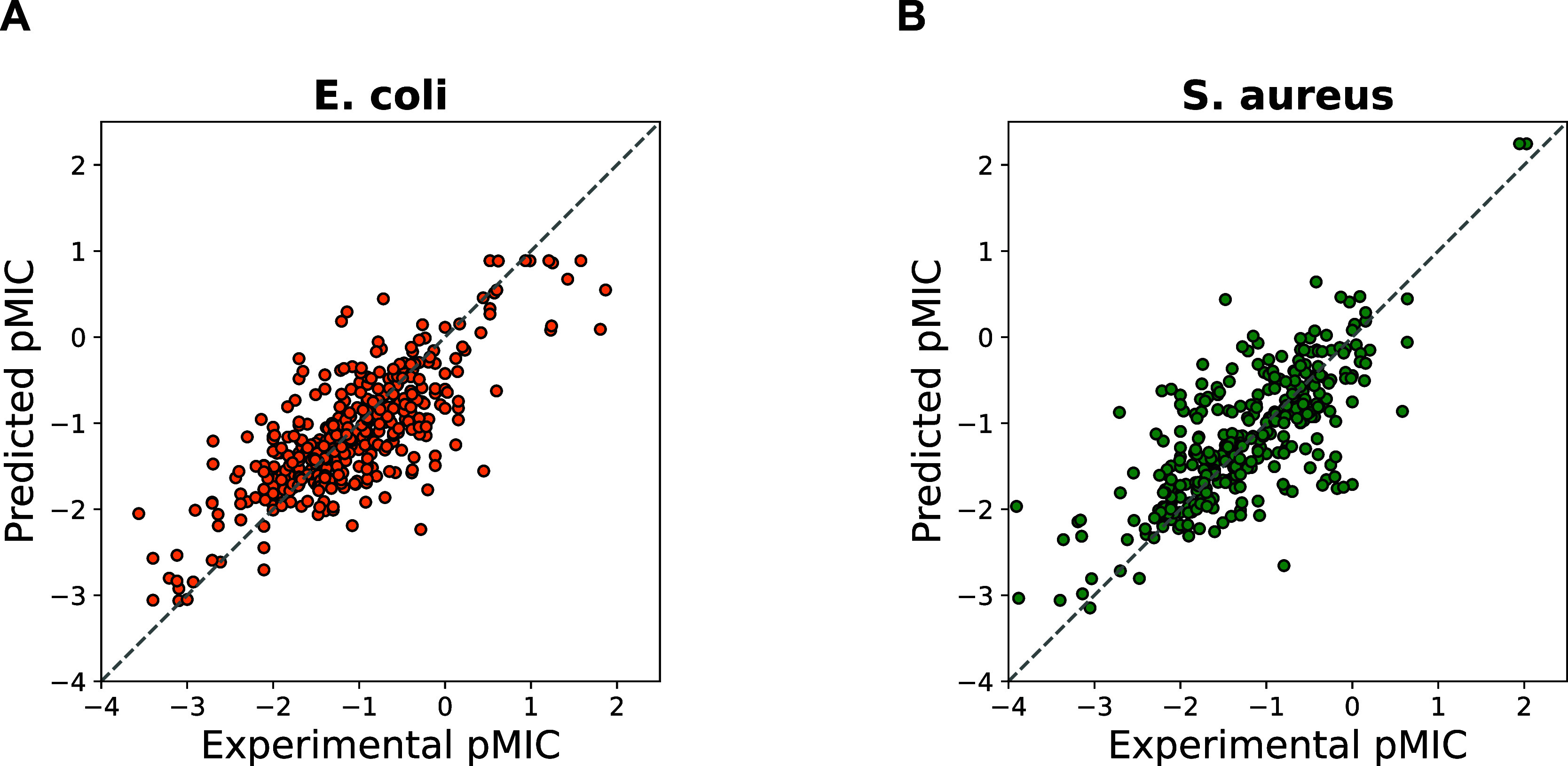
Correlation between experimental and predicted pMIC values in the test set using our models. (A) for *E. coli* and (B) for *S. aureus*.

The visual comparison of the ML and DL models in Figure 4 clearly demonstrates that our proposed model consistently outperforms other models across all metrics. This trend is evident for both EC and SA. The relatively short height of the box plots for our model indicates low variability in performance, suggesting robustness and reliability of the model. Figure 5 illustrates the correlation plot comparing the experimental pMIC and predicted pMIC values. Both models demonstrate a strong positive correlation between the experimental and predicted pMIC values, suggesting a good level of predictive power in the model. Overall, the superior performance of our BERT-based model demonstrates that pretraining with a large amount of unlabeled protein sequences enables BERT to effectively capture the general features of amino acid sequences. This provides significant advantages in the downstream task of predicting the biological activity of AMPs.

### 3.2 Ablation Study

**Figure 6:**
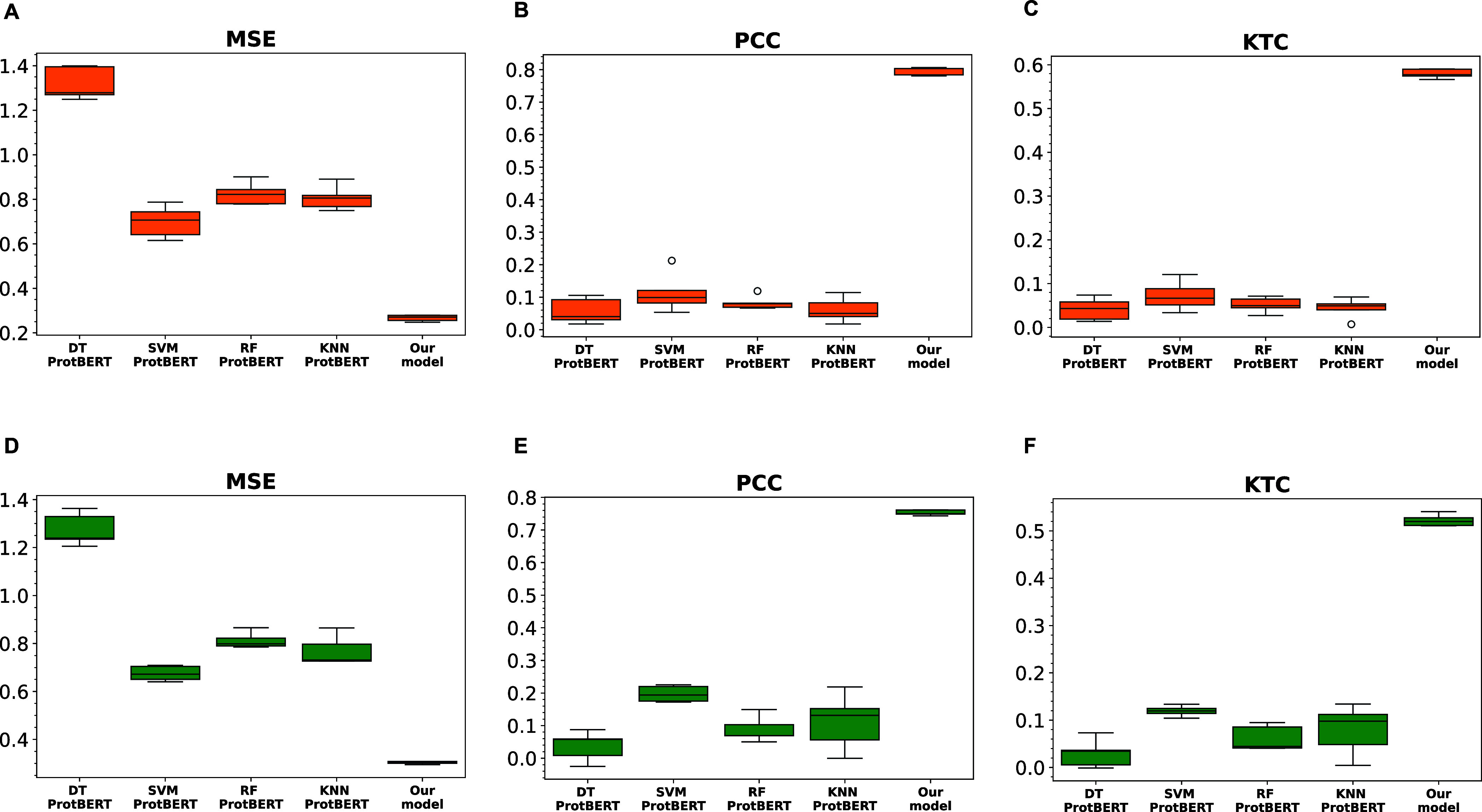
Ablation study. (A–C) for *E. coli* and (D–F) for *S. aureus*.

To demonstrate the effectiveness of transfer learning in predicting peptide activity, an ablation study was conducted. The original ProtBERT model was used as a feature extractor to convert peptide sequences into numerical vectors. These vectors were then used as input features for various traditional ML algorithms for regression analysis against quantitative activity labels. As shown in Table 4, the performance of the ML models using ProtBERT features is inferior to those using the more common peptide descriptors (QSOrder, CTDC, CTDT, CTDD, see Table 3). This result is surprising given that ProtBERT has been trained on a massive amount of protein sequence data. However, since AMP constitute only a small fraction of the entries in the protein sequence database, ProtBERT may not have comprehensively learnt the specific sequential patterns of AMPs. Nevertheless, based on the pretrained ProtBERT model and the use of transfer learning techniques, we successfully trained an improved AMP regression model that outperforms the best ML model for both EC and SA. This underlines the effectiveness of transfer learning as a valid strategy to train robust predictive models for challenging regression tasks. The visual comparison of all models in the abalation study in terms of MSE, PCC, and KTC are shown as a boxplot in Figure 6.

**Table 4:** Ablation Study

model	feature^a^	MSE	std^b^	PCC	std	KTC	std
*E. coli*							
SVM	ProtBERT	0.6987	0.0711	0.1136	0.0605	0.0723	0.0338
RF	ProtBERT	0.8251	0.0508	0.0833	0.0210	0.0515	0.0174
DT	ProtBERT	1.3183	0.0729	0.0575	0.0392	0.0416	0.0255
KNN	ProtBERT	0.8062	0.0546	0.0613	0.0378	0.0440	0.0231
BERT-AmPEP60	Embedding layer	**0**.**2664**	0.0141	**0**.**7955**	0.0121	**0**.**5797**	0.0104
*S. aureus*							
SVM	ProtBERT	0.6752	0.0309	0.1973	0.0246	0.1193	0.0110
RF	ProtBERT	0.8122	0.0331	0.0882	0.0390	0.0613	0.0266
DT	ProtBERT	1.2746	0.0679	0.0375	0.0451	0.0297	0.0296
KNN	ProtBERT	0.7693	0.0610	0.1116	0.0852	0.0793	0.0524
BERT-AmPEP60	Embedding layer	**0**.**3032**	0.0058	**0**.**7530**	0.0079	**0**.**5222**	0.0125

a Feature: Features extracted using the original ProtBERT model or our fine-tuned ProtBERT model.

b Std: standard deviation of the experiments with five replicates.

Therefore, it is not appropriate to use ProtBERT for feature extraction in the AMP regression task. However, we see that applying transfer learning significantly improves the model’s predictive performance, even surpassing the best ML algorithms. This confirms that the transfer learning approach is essential for effectively applying a protein language model to the AMP regression task. The results of the ablation study on the test set are shown in Figure 6.

### 3.3 Learning Curves Analysis across Different Data Set Sizes

**Figure 7:**
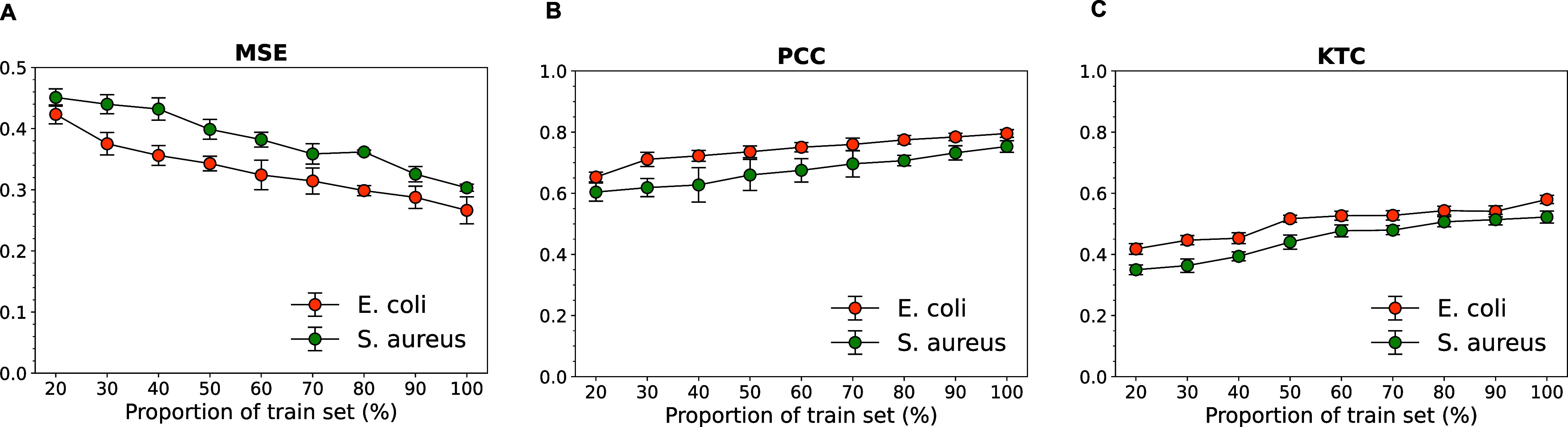
Learning curves of the our models with various train data scales. (A) MSE, (B) PCC, (C) KTC.

In predictive modeling, particularly working with limited data, it is crucial to assess the stability of the model across varying data sizes. To address this, we performed a learning curve analysis of the proposed method. Smaller data sets were generated by randomly selecting peptides from the train data set with reducing fractions from 90 to 20%. The generated data sets were used independently to train the models which are subsequently evaluated using the same test set as given in Table 1. As shown in Figure 7, models trained on reduced data sets exhibit worse performance across all metrics. Notably, the model trained on the full train set still performed slightly better than the model trained with 90% of the train data. This observation suggests that while the model’s performance continues to improve with increased data, it has not yet fully converged. Therefore, it is reasonable to expect that providing additional train data could further enhance the performance of the model.

### 3.4 Determination of Applicability Domain

Prediction models are developed based on a limited train data of labeled instances, and the assessment of the models is done by predicting unseen instances in a test set. Intuitively, the error rate for unseen instances that are dissimilar to the train data could be significantly higher than for those unseen instances that are similar to the train data. Therefore, the applicability of a model should be confined to the domain covered by the train set instances to avoid unreliable predictions with a high probability of error.^53^ Here, we consider the Euclidean distance of an instance to the centroid of the train data in the feature space as the similarity score^52^ and define the border of the applicability domain (AD) by setting a threshold D in eq 7 as follows:
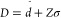
7where *d*– is the average of the similarity scores of all instances in the data set, σ denotes the standard deviation of similarity scores, and *Z* is an empirical parameter to control the coverages of AD. A range of *Z* from 0.5 to 2.0 with a step size of 0.5 has been tested, allowing for the evaluation of an incremental coverage area of the AD.

Table 5 shows the performance of our proposed models after defining an AD. For EC, the model with AD defined by *Z* = 0.5 achieved the best results, but it covers only 44% of the data. Switching from *Z* = 0.5 to *Z* = 1.0 doubled the amount of data that can be included for training. Therefore, to strike a balance between data coverage and model performance, we consider *Z* = 1.0 as the better choice. The model for EC with AD defined achieved an average MSE of 0.2447, PCC of 0.7980, and KTC of 0.5860. As for SA, coincidentally *Z* = 1.0 is also the best parameter and the model with AD defined achieved an average MSE of 0.2894, PCC of 0.7994, and KTC of 0.5544.

**Table 5:** Comparative Analysis of BERT-AmPEP60 with and without AD across Five Independent Test Experiments

model	AD	data coverage	MSE	PCC	KTC
*E. coli*					
BERT-AmPEP60	*Z* = 0.5	44%	0.2243	0.8044	0.5937
BERT-AmPEP60	***Z* = 1.0**	88%	**0.2447**	**0.7980**	**0.5860**
BERT-AmPEP60	*Z* = 1.5	92%	0.2635	0.7838	0.5897
BERT-AmPEP60	*Z* = 2.0	94%	0.2735	0.7812	0.5794
BERT-AmPEP60		100%	0.2664	0.7955	0.5797
*S. aureus*					
BERT-AmPEP60	*Z* = 0.5	30%	0.3177	0.7163	0.5088
BERT-AmPEP60	*Z* = 1.0	**77%**	0.2894	0.7994	0.5544
BERT-AmPEP60	*Z* = 1.5	93%	0.3049	0.7687	0.5406
BERT-AmPEP60	*Z* = 2.0	97%	0.3073	0.7539	0.5022
BERT-AmPEP60		100%	0.3032	0.7530	0.5222

### 3.5 Model Performance on External Data

To assess the generalizability of our final production models, we tested the EC and SA models on newly published sequences. A total of 82 AMPs were collected from recent research, including 17 AMPs against EC and 17 AMPs against SA generated using the AMP-Designer in the work of Wang et al.^54^ In addition, 34 AMPs against EC and 14 AMPs against SA generated with AMPd-Up were collected from the work of Li et al.^55^ These external sequences exhibit low sequence similarity with the train set of our models, with an average sequence identity below 20%. The distribution of sequence identity between these external sequences and the train sets is illustrated in Figure S3. For all these sequences, experimental antimicrobial assay data were available. Sequences with uncertain MIC values (e.g., >256 μM or >128 μM), were excluded from this analysis. Detailed information of these peptides can be found in Tables S2–S5.

**Figure 8:**
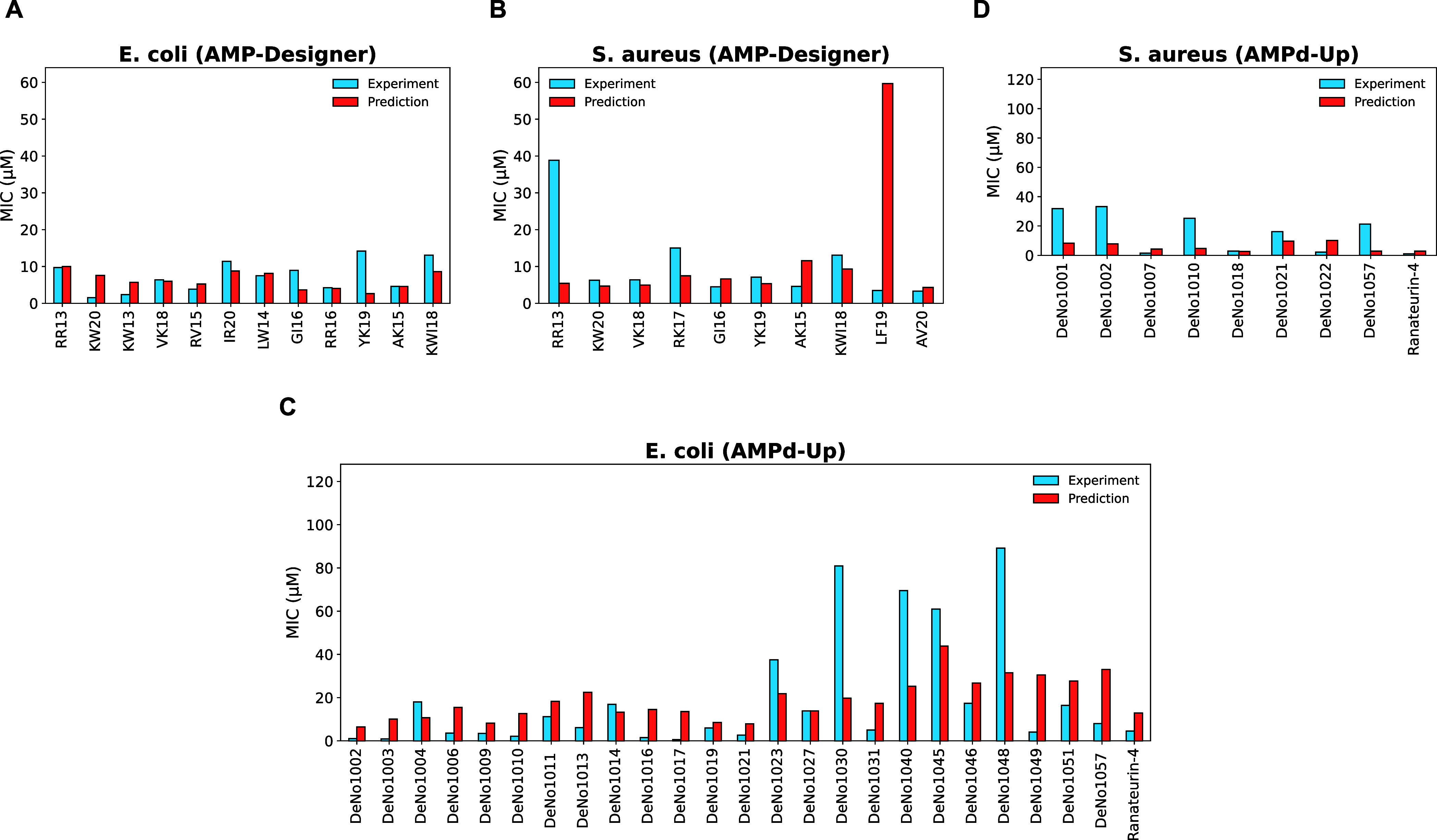
Comparison of the experimental and predicted MIC values (in μM) of the external data using the EC and SA models. (A, B) for the AMP-Designer sequences and (C, D for the AMPd-Up sequences).

For the AMP-Designer sequences, using the default *Z* = 1.0, 10 (out of 12) AMPs against EC and 9 (out of 10) AMPs against SA lie within the AD of our models. Using the same AD setting, 25 out of 34 AMPs against EC and 9 out of 14 AMPs against SA generated by AMPd-Up lie within the AD. Figure 8 shows the prediction results of our EC and SA models for the two external data within the AD. For AMPs from the AMP-Designer, both the EC and SA models performed well in predicting the MIC values. The EC and SA models achieved MSE of 0.1139 and 0.2610, respectively. For the AMPd-Up sequences, the models achieved an MSE of 0.3608 and 0.3215, respectively. We note that the AMPs from AMPd-Up have a high degree of sequence identity, but their activities are very different, which makes the prediction of these data extremely challenging.

The discrepancies between the predicted and experimental MIC values could stem from several factors related to data quality, model limitations, and the inherent complexity of AMPs. First, the variability in experimental data worth attention. MIC measurements are sensitive to variations in experimental conditions and protocols. However, condition data such as pH and ionic strength cannot be incorporated in the current model due to limited availability, therefore, only the sequence itself is learned. Second, discrepancies may also arise from the limited generalizability of the model. There are only thousands experimentally validated peptides with MIC values, which are limited. Therefore, the train data used to build the proposed models may not fully represent the diversity of peptides and bacterial targets that are necessary to accurately predict those peptides in the external data set. Finally, the biological complexity of peptides could pose a challenge in predicting their MIC values. Differences in the mechanisms of action of AMPs against different bacterial species may not be fully captured by the model.

## 4 Residue Importance Using Attention

Given that native AMPs are often biologically unstable and in some cases toxic to humans, continuous efforts have been made to develop analogs with improved biological properties and stability. These analogs typically consist of subsequences or mutations of a few residues from the wild-type sequence. A molecular biology technique commonly used to investigate the role of individual residues in a peptide to its biological activity is the single mutation scan, such as the alanine scan^56^ and lysine scan.^57^ By systematically substituting a residue with another amino acid of specific properties (such as alanine for nonreactivity and lysine for increased positive charge) and analyzing the change in experiment, the contribution of each amino acid residue can be quantified. This information serves as a valuable guide for rationally designing new AMPs with enhanced activity.

Given the need of rapid and accurate identification of residues from the sequence for understanding and improvement purposes, the attention mechanism in BERT is particularly relevant. The attention mechanism enables BERT-based prediction models to focus on the most pertinent parts of the input sequence when making predictions. These attention scores reflect the average importance of each token in the sequence based on the attention it receives from other tokens. Consequently, the attention scores could potentially serve as indicators of the importance of specific amino acids for the biological activity of peptides.

To evaluate our hypothesis, we made predictions on known AMPs and their analogs using the final prediction model. Since attention mechanism can identify higher-level features in deeper layers, we calculated the average attention that each amino acid receives from the other residues in the last attention layer. The average attention score is considered as the measure of importance of each amino acid in the sequence.^58^ As case studies, we selected Aurein 1.2 and its alanine scan analogs,^59^ along with LL-37-derived peptide fragment KR-12 and its alanine and lysine scan analogs.^60^ Both AMPs and their analogs have experimental MIC values which were converted into the unit of Molarity (μM) using eq 8, where *m* denotes the molecular mass of the peptide. We observed the relationship between the change in biological activity caused by a single mutation and the attention scores from the prediction model. In this way, we evaluated whether the attention mechanism recognizes the effects of individual amino acid residues on the biological and physicochemical properties of the peptide.
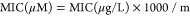
8

### 4.1 Alanine Scanning of Aurein 1.2

Aurein 1.2 (GLFDIIKKIAESF) is a short, naturally occurring AMP derived from the skin secretions of the Australian Southern Bell Frog *Litoria aurea*. It is an intriguing candidate for research and potential therapeutics due to its broad-spectrum activity against a diverse array of microbes, including bacteria, fungi, viruses, and even cancer cells. Numerous experimental studies have been conducted to elucidate the role of each amino acid residue in contributing to its antimicrobial efficacy. For example, Migorń et al.^59^ performed a single mutation scanning experiment to identify the residues crucial for aurein 1.2’s bioactivity.

In this case study, we predicted the MIC values for all single-mutation analogs of the aurein peptide and compared them with the experimental data by Migorń et al. This comparison would allow us to assess the sensitivity of our prediction models to single-point mutations and evaluate whether the attention mechanism is able to identify crucial residues that affect the biological activity of the peptide. Although the parent peptide, aurein 1.2, is included in the DBAASP, its analogs are not included in the data set, making it an excellent candidate for a case study to assess the predictive power of our models.

As presented in Table 6, the discrepancies between the predicted MIC values and the corresponding experimental data range from 5.5 to 285 μM. The three worst predictions are I6A, K7A and I9A. These mutations result in an 8-fold increase in the experimental MIC values, which was underestimated in the computational predictions as a reduction of only less than 2-fold. Meanwhile, the mutations that result in a decrease in MIC values i.e. enhance antimicrobial activity, particularly D4A, are also somewhat underestimated in the prediction. Overall, our EC model achieves a moderate predictive performance for the aurein analogs with an MSE (pMIC) of 0.2327 (see Table 8).

**Table 6:** Experimental and Predicted MIC Values of Aurein 1.2 and the Analogs against *E. coli*

peptide	sequence	experiment MIC (μg/mL)	mass (Da)	experiment^b^ MIC (μM)	prediction MIC (μM)	pred-exp. ΔMIC
aurein 1.2^a^	GLFDIIKKIAESF	64	1479.76	43.25	65.37	22.12
G1A	**A**LFDIIKKIAESF	128	1493.79	85.69	72.23	–13.45
L2A	G**A**FDIIKKIAESF	256	1437.68	178.06	86.53	–91.53
F3A	GL**A**DIIKKIAESF	128	1403.67	91.19	140.61	49.42
D4A	GLF**A**IIKKIAESF	8	1435.75	5.57	37.82	32.24
I5A	GLFD**A**IKKIAESF	256	1437.68	178.06	83.72	–94.34
I6A	GLFDI**A**KKIAESF	512	1437.68	356.13	85.43	–270.70
K7A	GLFDII**A**KIAESF	>512	1422.67	359.89	82.11	–277.78
K8A	GLFDIIK**A**IAESF	128	1422.67	89.97	54.42	–35.55
I9A	GLFDIIKK**A**AESF	512	1437.68	356.13	70.94	–285.19
E11A	GLFDIIKKIA**A**SF	16	1421.72	11.25	48.37	37.12
S12A	GLFDIIKKIAE**A**F	64	1463.76	43.72	49.18	5.46
F13A	GLFDIIKKIAES**A**	128	1403.76	91.19	199.15	107.96

a Sequence in the train set.

b *E. coli* ATCC 25922.

**Figure 9:**
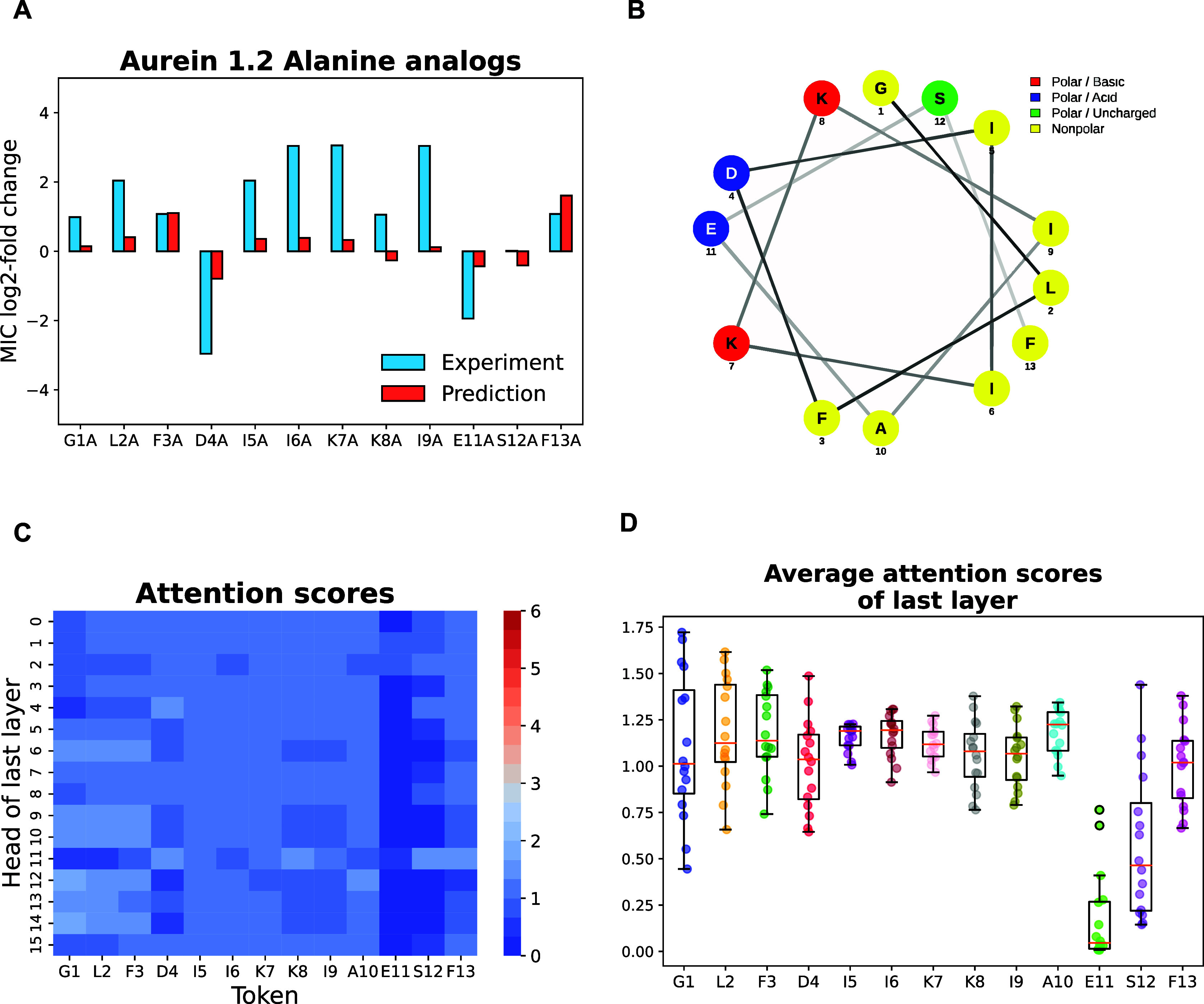
Case study of aurein 1.2 and its mutation analogs against *E. coli*. (A) Comparison of experimental and predicted Log_2_-fold changes of MIC values. (B) Helical wheel projection of aurein 1.2 (generated by NetWheels).^100^ (C) Heatmap of attention scores across the heads of the final BERT layer. (D) Average attention scores for each amino acid residue in aurein 1.2.

For a more intuitive comparison, we plotted the log2-fold activity changes resulting from alanine mutations in Figure 9. A positive value indicates a reduction in antimicrobial activity compared to the native peptide, while a negative value signifies an increase. We note that the direction of change for both experimental and prediction results is consistent across most analogs, with the exception of K8A and S12A. Importantly, substitutions D4A and E11A were identified as potential substitutions that may improve the potency of the peptide, although the predicted magnitude of change is underestimated.

Interestingly, these two residues, D4 and E11, are located on the same face of the helix as the two lysine residues (see Figure 9B). According to the authors of,^59^ while mutation of these two residues to a nonpolar amino acid might impair the amphipathic nature of the helix, their substitutions increase the net positive charge of the peptide. This reduces the electrostatic repulsion, a key factor in improving membrane adsorption of the peptide. Although S12A shows no difference in antimicrobial activity against *E. coli*, previous findings indicate that it exhibits increased hydrophobicity and biostability of the peptide, along with stronger antimicrobial activity against *S. aureus*.

The attention heatmap presented in Figure 9C illustrates the attention scores of each head in the last layer of the BERT encoder for the aurein peptide. In BERT’s multihead attention mechanism, multiple heads allow the model to simultaneously attend to information from different representational subspaces. Each head computes distinct attention scores for each residue, reflecting the degree of importance or attention the model assigns to each position in the sequence. As shown in Figure 9D, the average attention scores for most amino acid residues in aurein are similar, except for residues E11 and S12, which receive notably lower level of attention. This suggests that E11 and S12 may be less critical for the peptide’s bioactivity compared to the other residues. Consequently, E11 and S12 may serve as good candidates for substitutions aimed at enhancing the peptide’s bioactivity.

### 4.2 Alanine and Lysine Scanning of KR-12

The human antimicrobial peptide LL-37, belongs to cathelicidin family, displays broad spectrum antibacterial activity and has been considered as a potential alternative to conventional antibiotics. Despite its effectiveness, LL-37 proved to have hemolysis for eukaryotic cells, prompting the exploration of its derivatives.^61^ A notable derivative is KR-12 (residues 18–29 of LL-37, KRIVQRIKDFLR), it possess antimicrobial activities and only caused ∼10% hemolysis of erythrocytes at 100 μM.^62^ In the study by Gunasekera et al.,^60^ experimental alanine and lysine scans on KR-12 were conducted, yielding two series of 12 and 10 analogs, respectively.

Using our BERT-based EC prediction model, we obtained the predicted MIC values for all alanine and lysine analogs, which are compared to the corresponding experimental data in Table 7. We should point out that since KR-12 and its analogs are included in the DBAASP database, these sequences are part of the train set for model construction in the beginning of this study (indicated with a ∗ in the result table). Consequently, the prediction results of KR-12 analogs are pretty accurate, with maximum absolute errors of less than 15 μM.

**Table 7:** Experimental and Predicted MIC Values of KR-12 Analogs against *E. coli*

peptide	sequence	mass (Da)	experiment^b^ MIC (μM)	prediction MIC (μM)	pred-exp. MIC ΔMIC
KR-12^a^	KRIVQRIKDFLR	1571.93	2.5	2.82	0.32
K1A^a^	**A**RIVQRIKDFLR	1514.84	40	47.80	7.80
R2A	K**A**IVQRIKDFLR	1486.82	40	46.50	6.50
I3A^a^	KR**A**VQRIKDFLR	1529.85	5	4.40	–0.60
V4A	KRI**A**QRIKDFLR	1543.88	5	5.92	0.92
Q5A^a^	KRIV**A**RIKDFLR	1514.88	5	7.31	2.31
R6A	KRIVQ**A**IKDFLR	1486.82	40	46.36	6.36
I7A	KRIVQR**A**KDFLR	1529.85	40	32.58	–7.42
K8A^a^	KRIVQRI**A**DFLR	1514.84	40	30.54	–9.46
D9A	KRIVQRIK**A**FLR	1527.92	1.25	1.54	0.29
F10A^a^	KRIVQRIKD**A**LR	1495.83	10	14.81	4.81
L11A^a^	KRIVQRIKDF**A**R	1529.85	20	16.60	–3.40
R12A^a^	KRIVQRIKDFL**A**	1486.82	40	55.12	15.12
R2K^a^	K**K**IVQRIKDFLR	1543.92	5	4.68	–0.32
I3K^a^	KR**K**VQRIKDFLR	1586.95	1.25	0.99	–0.26
V4K	KRI**K**QRIKDFLR	1600.97	2.5	1.41	–1.09
Q5K^a^	KRIV**K**RIKDFLR	1571.98	1.25	0.97	–0.28
R6K	KRIVQ**K**IKDFLR	1543.92	5	6.85	1.85
I7K	KRIVQR**K**KDFLR	1586.95	20	28.15	8.15
D9K	KRIVQRIK**K**FLR	1585.02	0.626	1.30	0.68
F10K	KRIVQRIKD**K**LR	1552.93	5	6.26	1.26
L11K	KRIVQRIKDF**K**R	1586.95	10	10.76	0.76
R12K	KRIVQRIKDFL**K**	1543.92	5	5.84	0.84

a Sequence in the train set.

b *E. coli* ATCC 25922.

**Figure 10:**
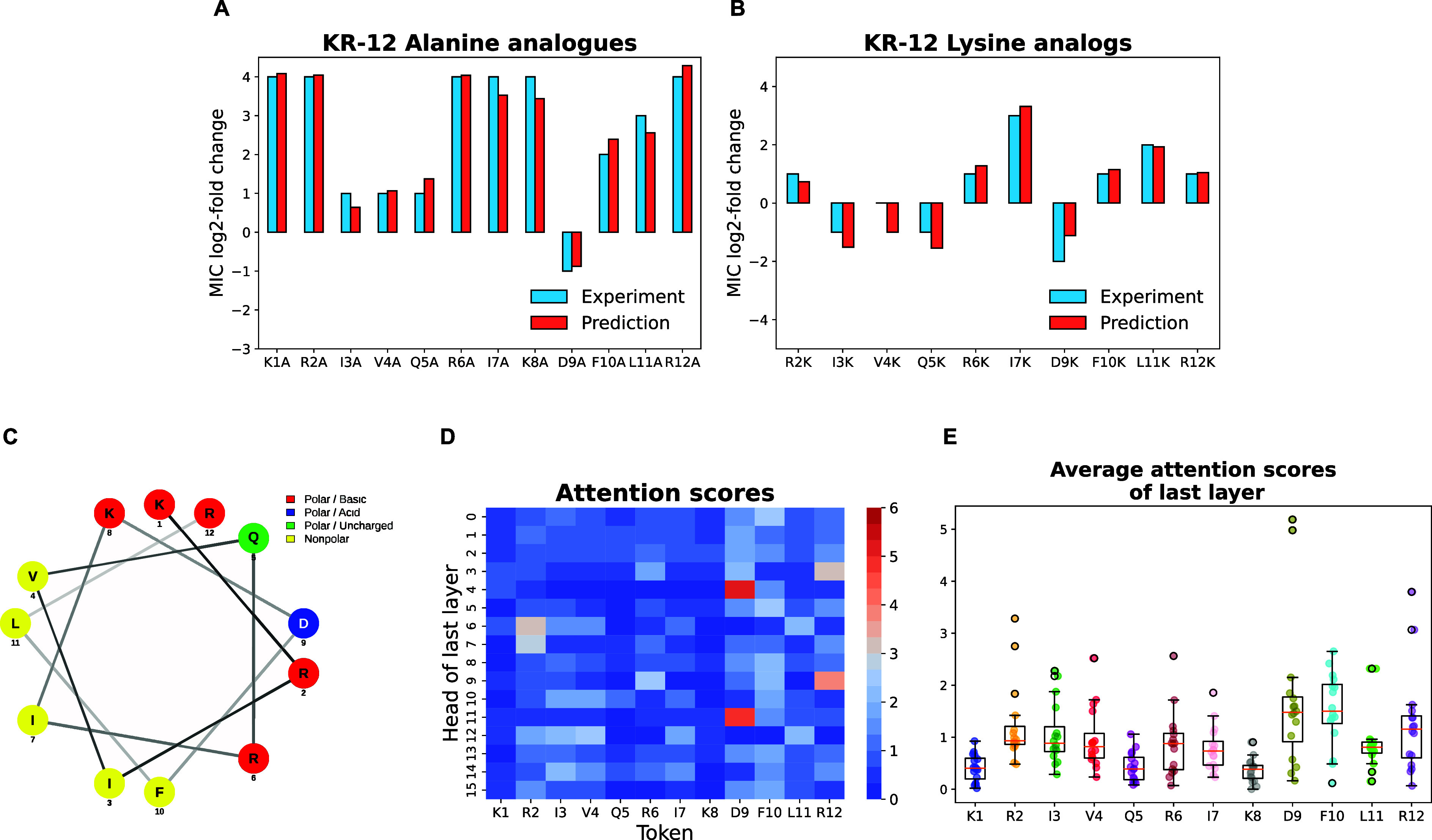
Case study of KR12 and its mutation analogs against *E. coli*. Comparison of experimental and predicted Log_2_-fold changes of MIC values for (A) alanine scan analogs and (B) lysine scan analogs. (C) Helical wheel projection of KR-12 (generated by NetWheels). (D) Heatmap of attention scores across the heads of the final BERT layer. (E) Average attention scores for each amino acid residue in KR-12.

Figure 10A,B illustrate a comparison of the log_2_-fold changes in MIC upon mutation observed in the experimental and computational studies. In the alanine scan, most mutations of KR-12 resulted in a reduction of activity ranging from 1 to 4 log_2_-fold changes in MIC. An exception was observed with the mutation of Asp9 to alanine (D9A), which is an increase in the peptide’s activity. Here, computational predictions align excellently with the experimental findings. On the other hand, despite greater discrepancies in predicted MIC values for the lysine scan, the trend of changes in activity remains consistent, indicating the predictability of mutational impact at least qualitatively.

Several residues (I3, V4, Q5, and D9) have been identified here as potential candidates for mutations that improve activity. Specifically, D9 (which resides on the positively charged surface of the helical KR-12, see Figure 10C) emerges as a candidate for substitution in both alanine and lysine scans, suggesting its unfavorable role. It is known that an overall positive charge is important for the bioactivity of AMPs,^63^ therefore replacing the negatively charged Asp9 with neutral alanine or positively charged lysine resulted in increased activity against EC. Figure 10D depicts the heatmap of attention scores across the heads of the last layer of the BERT model. From the heatmap analysis, it is evident that D9 received notably high attention scores in head 4 and head 11. Consequently, this led to D9 having the highest average attention score (see Figure 10D). In this context, the crucial amino acid refers to the residue that impedes or reduces the activity of the peptide. When this residue is replaced with another amino acid, it results in an enhancement of the peptide’s activity.

Interestingly, this result is in stark contrast to that of aurein 1.2. The two residues E11 and S12 of the aurein peptide, in which the alanine mutation resulted in increased antimicrobial activity, have relatively low attention scores. The two different observations may indicate that amino acids with extreme maximum and minimum attention values should be considered and that peptides with stronger antimicrobial activity can be obtained by replacing these amino acids with other residues. By focusing this task on a smaller set of residues, the BERT-based model proposed here would facilitate their experimental tractability.

In general, helicity, hydrophobicity and aggregation propensity play an essential role in the microbial specificity of AMPs.^64^ Appropriate modification of the sequences would improve their bioactivity. As shown in Table 8, prediction of analogs of aurein 1.2 and KR-12 resulted in low MSE values, high PCC and KTC values. Sequences that existed in the train set were not counted in the performance metrics. These case studies demonstrate the potential utility of the proposed model as an effective evaluation tool for the design of artificial AMPs by modification of known AMPs or even de novo design. In addition, further observations should be made to provide a reliable explanation for more applications of attention mechanism-based models. We also present the heatmaps of attention scores and scatter plots of average attention scores for computational mutagenesis of aurein 1.2 in Figures S4 and S5 and KR-12 in Figures S7–S9, these attention analysis could help to identify potential residues, where further mutation will enhance the peptide bioactivity.

**Table 8:** Performance of our Model in Predicting the pMIC Values of AMP Analogs for *E. coli*

AMP	analogs	MSE	PCC	KTC
KR-12	alanine scan	0.0059	0.9954	0.8366
KR-12	lysine scan	0.0312	0.9348	0.9258
aurein 1.2	alanine	0.2327	0.5274	0.4187

## 5 Discussion and Conclusions

In this work, we applied the transfer learning strategy to the pretrained protein language model, ProtBERT, constructing deep regression models for predicting the MIC values of AMPs for EC and SA. Doing this, our aim is to provide specific, reliable, and explainable predictions for the antimicrobial activity of peptides. The ProtBERT model has been pretrained with billions of protein sequences from the UniRef100 database. This enables the ability to extract sequence information from the input amino acid sequences. For the downstream regression task, the CLS token from ProtBERT is utilized to encapsulate the general features of the given sequence.

We trained the deep regression models against two clinically relevant bacterial species EC and SA using the data curated from the DBAASP database. The two bacteria were deliberately chosen. On the one hand, they are the two most-studied bacteria for which sufficient data is available, and on the other hand, they are considered by the World Health Organization to be critical, priority pathogens due to their tendency to develop antibiotic resistance, which makes the treatment of infections increasingly difficult.^65^

The comprehensive evaluation of the prediction models using the test sets demonstrates that our regression models outperform traditional ML models and a previously established regression model of EC.^38^ Specifically, our model for EC achieved an MSE of 0.2664, PCC of 0.7955 and KTC of 0.5797, which is an improvement of 17, 3.2, and 5%, respectively, compared to the second best model MBC-Attention, and an improvement of 22.5, 8.2, and 13%, respectively, to the RF model. Meanwhile, the AMP prediction for SA proved to be more challenging. Nevertheless, our model achieved an MSE of 0.3032, PCC of 0.7530 and KTC of 0.5222. These results represent improvements of 14.5, 6.3, and 6.0%, respectively, compared to the MBC-Attention model and 19.0, 9.5, and 14.1%, respectively, to the RF model.

As a step toward the explainability of DL models, we investigated how biological activity can be correlated with input sequences using the attention mechanism. Several studies have shown that attention scores assigned to individual residues can be used to identify crucial residue-based patterns in the sequence.^66,42^ In this work, we investigated the correlation of attention scores with the activity prediction of AMP in two case studies for which experimental mutagenesis results were available. In the alanine scan of aurein 1.2, substitution of alanine for the residue with the lowest attention score led to an improvement in bioactivity. This is consistent with our intuition that the least attended residues probably contribute less to the biological function of the molecule. In contrast, in the alanine and lysine scans of KR-12, the residue with the highest attention score was found to improve bioactivity upon mutation. The two contrasting observations suggest that residues with extreme attention scores deserve special attention. Appropriate substitution of these residues could lead to an improvement in bioactivity.

Since quantitative prediction models for AMPs are rare, the developed BERT-AmPEP60 models are instrumental in providing precise estimation of the potential antimicrobial activity of novel sequences. To facilitate utility, a user-friendly web server for predicting AMPs using BERT-AmPEP60 are made online. Researchers focused on combating antimicrobial resistance can submit sequences to the Web site, which will then generate predictions of MIC values (in units of μM) against EC and SA. The predicted MIC values allow researchers to rank and prioritize the peptides based on their potential antimicrobial potency so that the sequences with the lower MIC values can be identified for experimental validation. This approach streamlines the screening process and saves both time and resources. Furthermore, the attention scores from our proposed models can highlight specific amino acids or motifs that are critical for antimicrobial activity, providing valuable insights into sequence features that can guide optimization efforts. Researchers can use this information to design and test modifications and iteratively refine peptides to improve their potency and properties. By integrating BERT-AmPEP60 into their workflows, researchers can rapidly identify and optimize promising antimicrobial candidates, accelerating the discovery process.

Although the experimental results are encouraging, there are still limitations and potential areas for model improvements. First, the vast prevalence of non-AMP peptides poses a challenge for regression models. As certain peptides might be overestimated in their activities, especially those with high similarity to known AMPs or those at the borderline between active and inactive AMPs. To mitigate this problem, the applicability domain of our models has been developed to ensure reliable performance and minimize uncertain predictions. Furthermore, using a robust AMP classifier could significantly reduce false positives, as classification models are more effective in distinguishing between active and inactive AMPs. Regarding data preprocessing, for those sequences with multiple MIC values due to different reference sources and experimental setting, we took the average as the activity value in this work. When the standard deviation of multiple MIC values of one sequence is large, a deeper investigation into the reference sources could be conducted to further improve quality of the data. Second, one major limitation in AMP prediction is the relatively small data size compared to the large protein data sets used for pretraining language models. This presents a challenge when attempting to fine-tune a large pretrained language model for specific downstream tasks. One strategy is to add domain transfer learning before the specific downstream task, which can improve domain adaptation of the large language model to the downstream task domain.^67^

The development of novel antimicrobial agents such as AMPs is critical to counter the threat of increasing antibiotic resistance. To discover potent AMPs, two main strategies can be explored: first, rational design based on existing natural AMPs as template, and second, de novo design. For the latter, innovative methods such as generative algorithms, which have been successfully applied to image and text generation, are promising. It is expected that these techniques will allow a thorough exploration of the sequence space, which is otherwise a formidable task with the traditional evolutionary algorithms commonly used in peptide design.

## Data Availability

All peptide data used in this study comes from DBAASP^40^ (https://dbaasp.org/home). The data and data preparation scripts for reproducing the experiments can be downloaded from https://github.com/janecai0714/AMP_regression_EC_SA. The final prediction models trained in this study are also available in the previous link. The web server to directly access this prediction method is at https://app.cbbio.online/ampep/home.

## Supporting Information Available

Distributions of sequence identity between the train set and the test set; convergence curve of our model; distributions of sequence identity between the AMPs from external data and the train set; heatmap of attention scores across the heads of the final layer of our EC model for aurein 1.2 alanine mutation analogs; average attention scores for each amino acid residue in aurein 1.2 alanine mutation analogs by EC model; heatmap of attention scores across the heads of the final layer of our EC model for KR-12 alanine mutation analogs; average attention scores for each amino acid residue in KR-12 alanine mutation analogs by EC model; heatmap of attention scores across the heads of the final layer of our EC model for KR-12 lysine mutation analogs; average attention scores for each amino acid residue in KR-12 lysine mutation analogs by EC model; comparative analysis of machine learning and deep learning models for predicting MIC of peptides across five independent validation experiments; experimental and predicted MIC values of AMPs against *E. coli* from AMP-Designer; experimental and predicted MIC values of AMPs against *S. aureus* from AMP-Designer; experimental and predicted MIC values of AMPs against *E. coli* from AMPd-Up; and experimental and predicted MIC values of AMPs against *S. aureus* from AMPd-Up (PDF)
