# Supplementary material for: BERT-AmPEP60: A BERT-Based Transfer Learning Approach to Predict the Minimum Inhibitory Concentrations of Antimicrobial Peptides for Escherichia coli and Staphylococcus aureus
Source: J Chem Inf Model. 2025 Mar 14;65(7):3186–202. doi: 10.1021/acs.jcim.4c01749 (PMC12004541; doi:10.1021/acs.jcim.4c01749)
Supplement: Supplementary file 1 — ci4c01749_si_001.pdf [file ci4c01749_si_001.pdf]

BERT-AmPEP60: A BERT-based Transfer Learning Approach to Predict the Minimum Inhibitory Concentrations of Antimicrobial Peptides for *Escherichia coli* and *Staphylococcus aureus*

Jianxiu Cai,[1,2] Jielu Yan,[2,3] Chonwai Un,[4]
Yapeng Wang,[1,*], François-Xavier Campbell-Valois,[5,6,7,*]
Shirley W. I. Siu[8,*]

[1]Faculty of Applied Sciences,
Macao Polytechnic University, Rua de Luís Gonzaga Gomes, Macau SAR, China
[2]Insitute of Science and Environment,
University of Saint Joseph, Rua de Luís Gonzaga Gomes, Macau SAR, China
[3]School of Computer Science,
Chongqing University, Shapingba, Chongqing, China
[4]T-Rex Technology HK Limited,
5/F, Building 5E, 5 Science Park East Avenue, Hong Kong Science Park, Hong Kong
[5]Host-Microbe Interactions Laboratory,
Center for Chemical and Synthetic Biology, Department of Chemistry and Biomolecular Sciences,
University of Ottawa, Ottawa, Canada
[6]Centre for Infection, Immunity, and Inflammation,
University of Ottawa, Ottawa, Canada
[7]Department of Biochemistry, Microbiology and Immunology
University of Ottawa, Ottawa, Canada
[8]Centre for Artificial Intelligence Driven Drug Discovery, Faculty of Applied Sciences,
Macao Polytechnic University, Rua de Luís Gonzaga Gomes, Macau SAR, China

[*]To whom correspondence should be addressed;
E-mail: yapengwang@mpu.edu.mo; fcampbel@uOttawa.ca; shirleysiu@mpu.edu.mo.

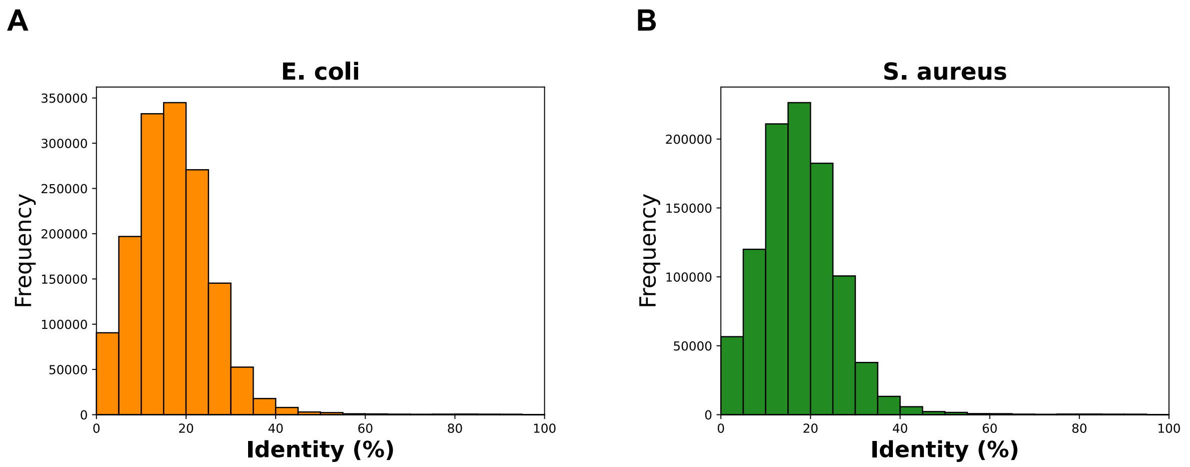

Figure S1: Distributions of sequence identity between the train set and the test set. **A** for *E. coli* and **B** for *S. aureus*

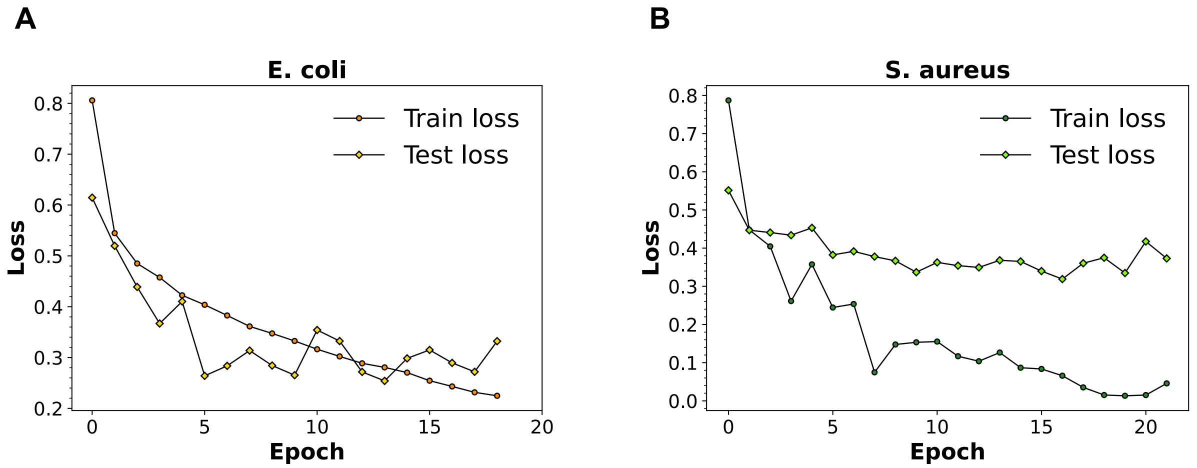

Figure S2: Convergence curve of our model. **A** for *E. coli* and **B** for *S. aureus*

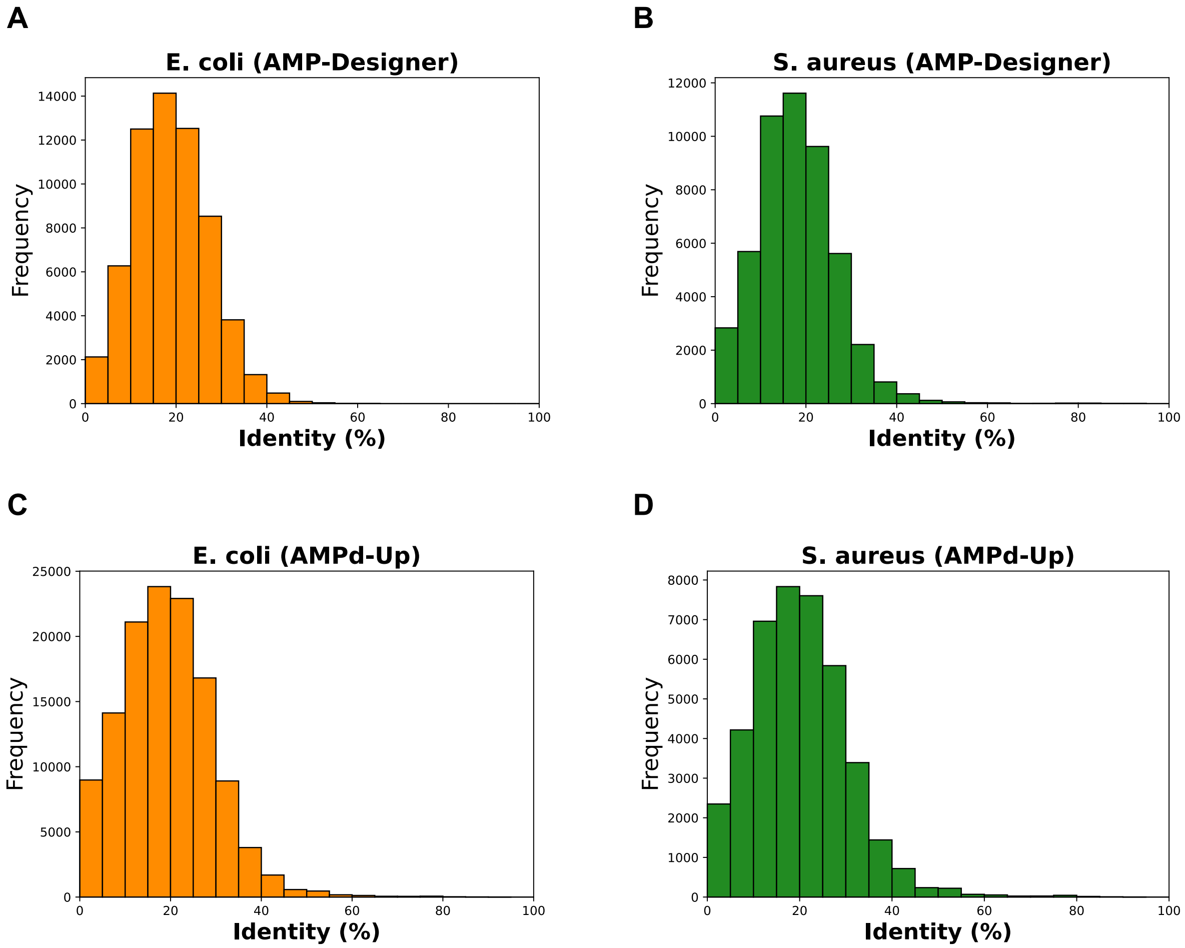

Figure S3: Distributions of sequence identity between the AMPs from external data and the train set. **A** for *E. coli* and **B** for *S. aureus*

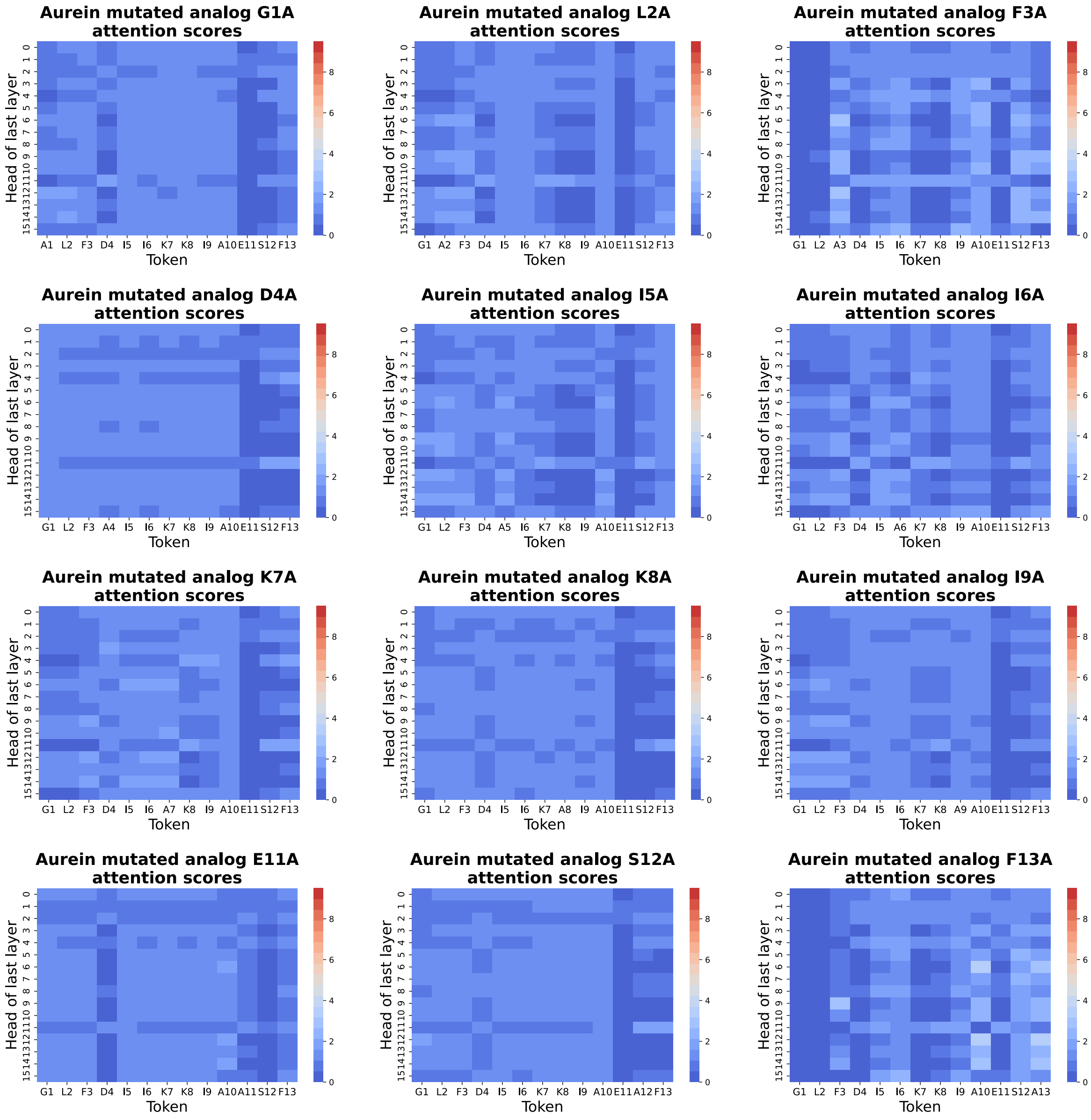

Figure S4: Heatmap of attention scores across the heads of the final layer of our EC model for aurein 1.2 alanine mutation analogs.

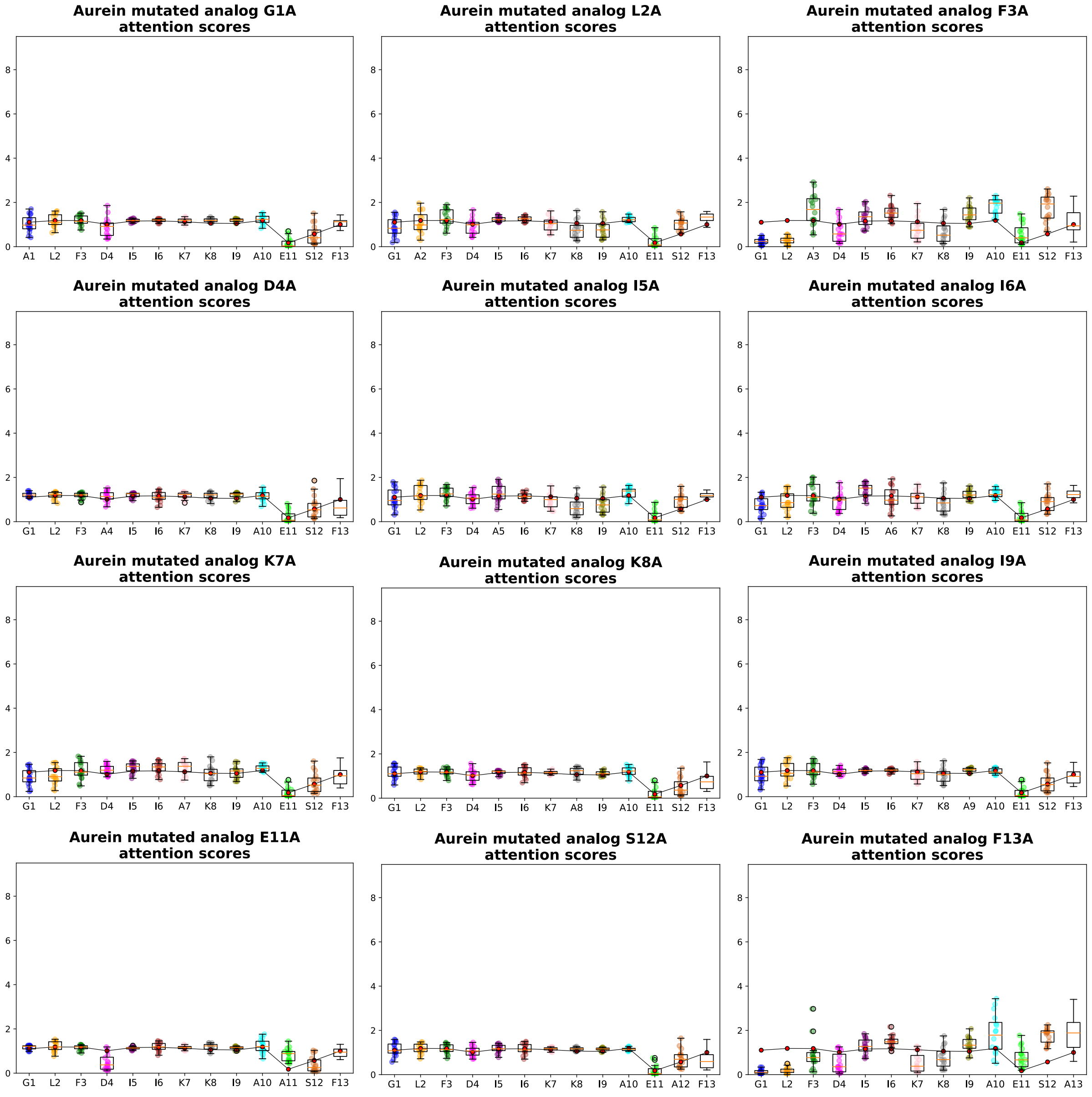

Figure S5: Average attention scores for each amino acid residue in aurein1.2 alanine mutation analogs. Scores are calculated by our EC model.

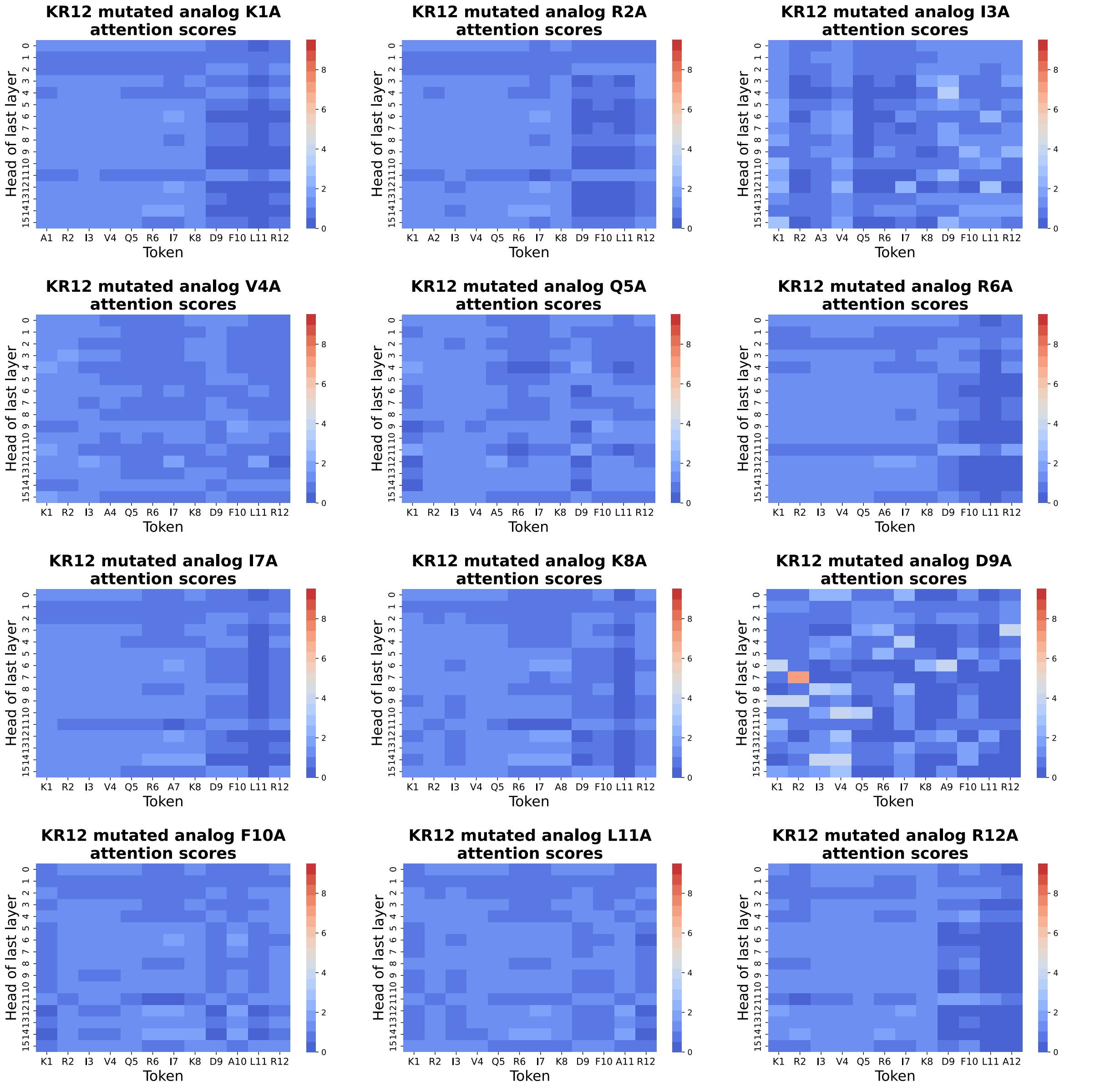

Figure S6: Heatmap of attention scores across the heads of the final layer of our EC model for KR-12 alanine mutation analogs.

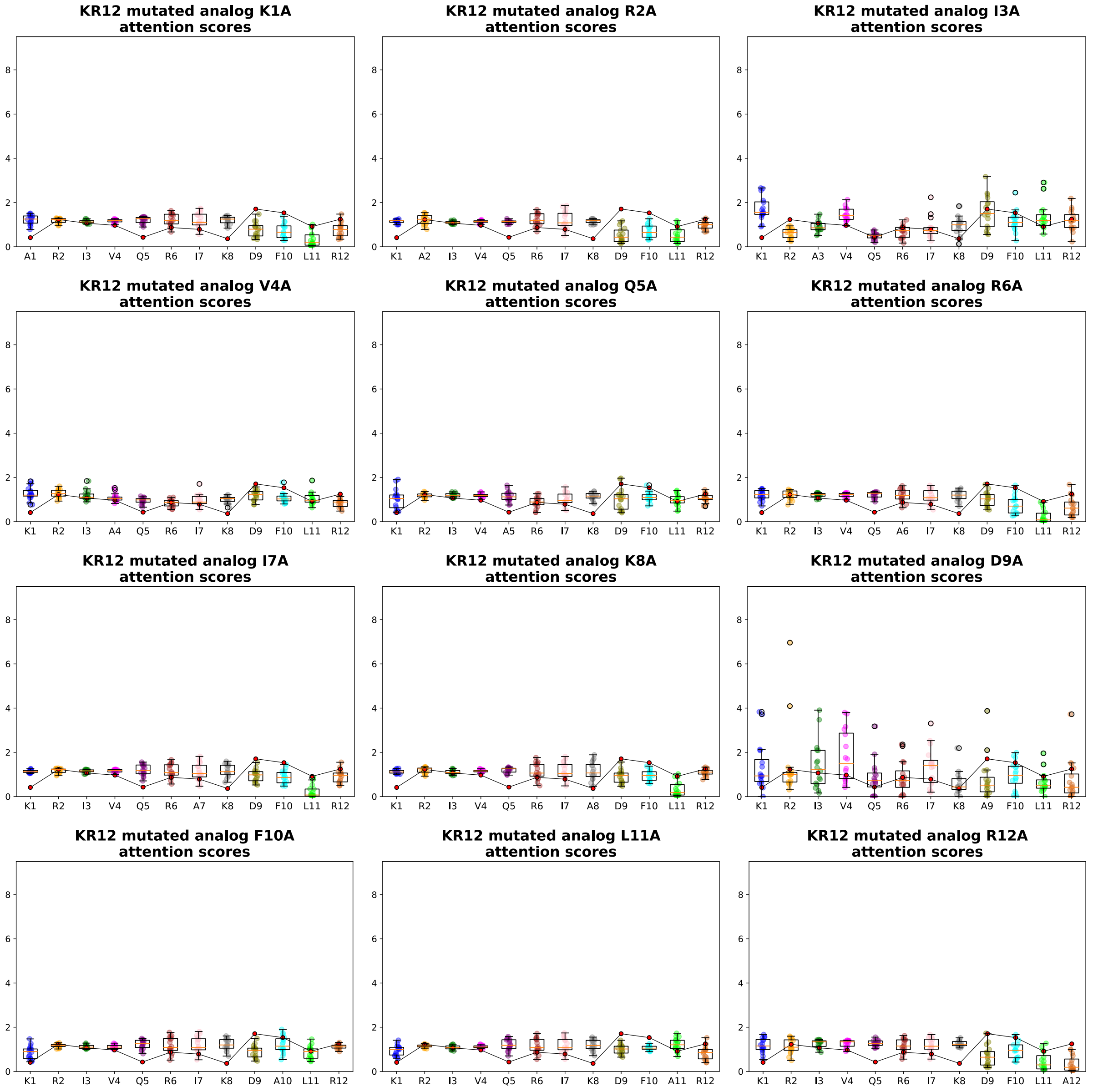

Figure S7: Average attention scores for each amino acid residue in KR-12 alanine mutation analogs. Scores are calculated by our EC model.

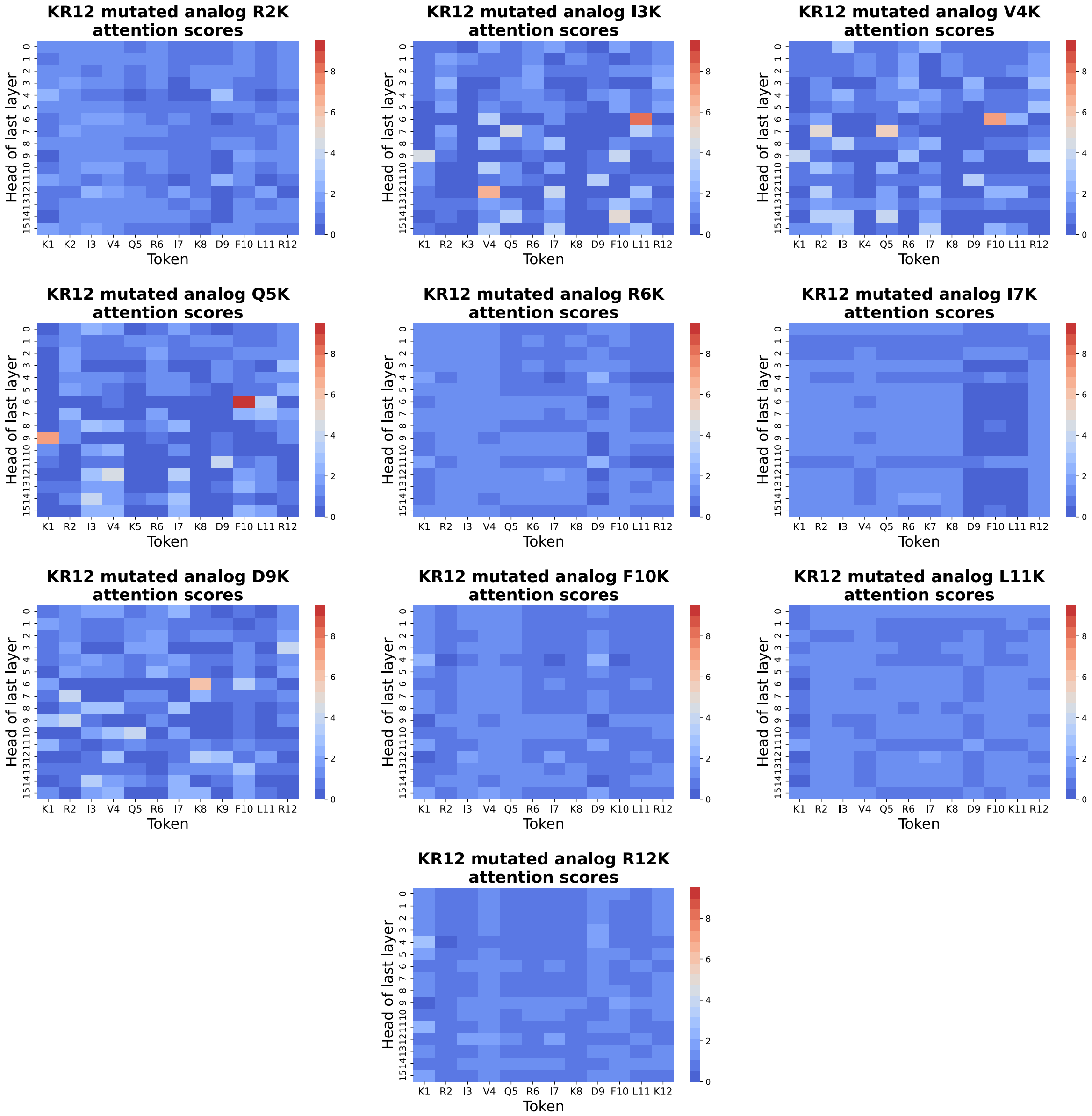

Figure S8: Heatmap of attention scores across the heads of the final layer of our EC model for KR-12 lysine mutation analogs.

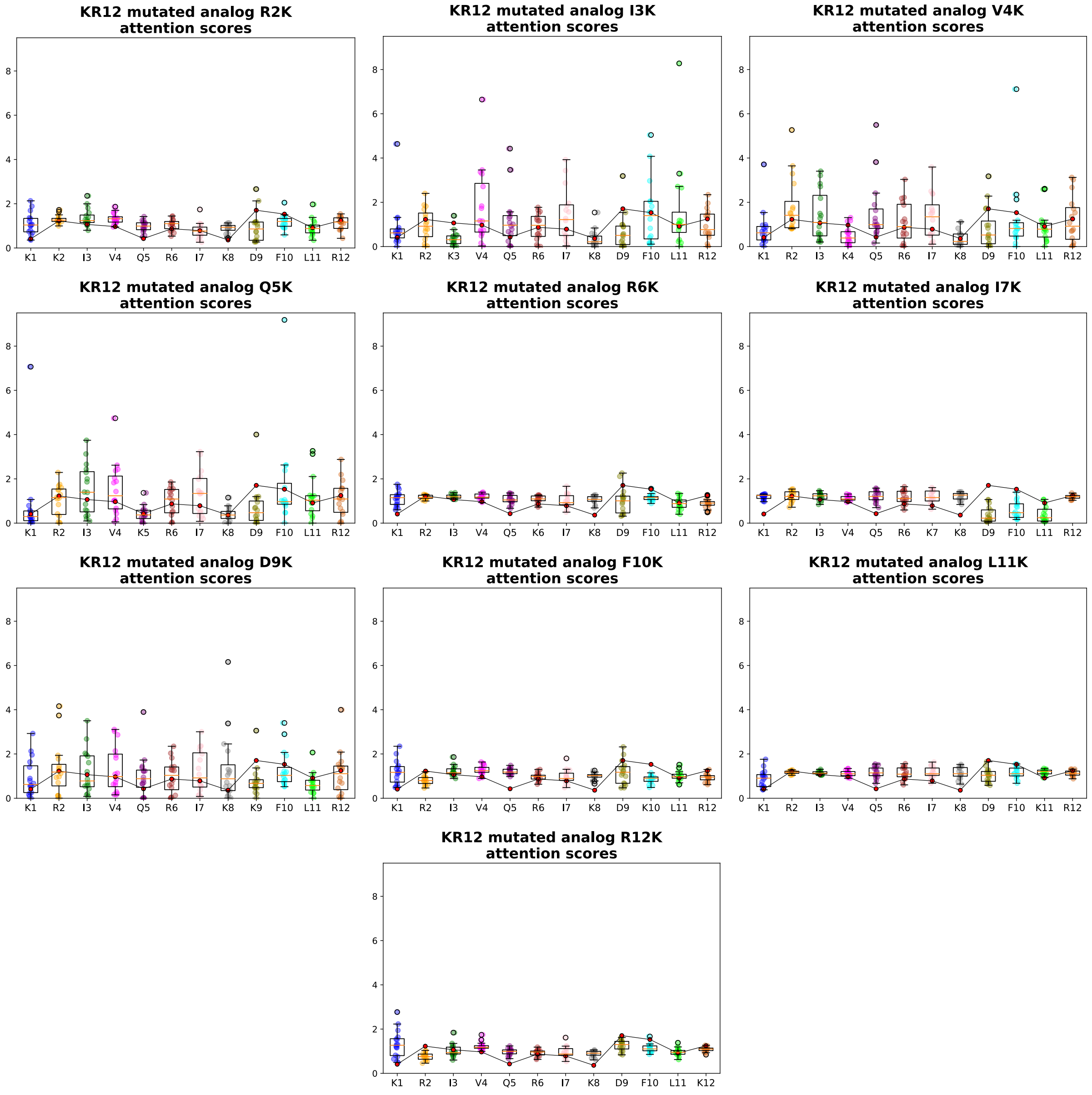

Figure S9: Average attention scores for each amino acid residue in KR-12 lysine mutation analogs. Scores are calculated by our EC model.

Table S1: Comparative analysis of machine learning and deep learning models for predicting MIC of peptides across five independent validation experiments.

Model	Feature[a]	MSE	std[b]	PCC	std	KTC	std
E. coli							
DT	QSOrder	0.6788	0.0505	0.5502	0.0242	0.3641	0.0300
SVM	QSOrder	0.3852	0.0391	0.6891	0.0268	0.4540	0.0163
RF	CTDD	0.3696	0.0381	0.7160	0.0266	0.4729	0.0210
KNN	QSOrder	0.4073	0.0521	0.6518	0.0347	0.4217	0.0182
MBC	Feature combination[c]	0.3422	0.0197	0.7354	0.0279	0.5271	0.0381
MBC-Attention	Feature combination	0.3305	0.0186	0.7679	0.0237	0.5536	0.0167
BERT-AmPEP60	Embedding layer	**0.2644**	0.0340	**0.7975**	0.0144	**0.5997**	0.0202
S. aureus							
DT	CTDD	0.7433	0.0347	0.4496	0.0580	0.2734	0.0468
SVM	QSOrder	0.4641	0.0367	0.5251	0.0297	0.3920	0.0192
RF	CTDD	0.3667	0.0202	0.6839	0.0247	0.4616	0.0371
KNN	QSOrder	0.4592	0.0282	0.5970	0.0308	0.3858	0.0233
MBC	Feature combination	0.3451	0.0459	0.7192	0.0425	0.4930	0.0295
MBC-Attention	Feature combination	0.3359	0.0587	0.7189	0.0469	0.5167	0.0399
BERT-AmPEP60	Embedding layer	**0.3072**	0.0188	**0.7510**	0.0286	**0.5322**	0.0158

[a]Feature: Protein sequence descriptors or embeddings from the fine-tuned ProtBERT model.

[b]std: standard deviation of the experiments with five replicates.

[c]Feature combination: the best-14 feature types based on the PseKRAAC (Pseudo K-tuple Reduced Amino Acids Composition) and QSORder.

Table S2: Experimental and predicted MIC values of AMPs against *E. coli* from AMP-Designer

Peptide	Sequence	Experiment MIC (μg/mL)	Mass (Da)	Experiment† MIC (μM)	Prediction MIC (μM)
RR13	`RRWFKIRMAAKLA`	16	1647.05	9.71	10.00
KW20	`KWRKVRAKFWRKWLAGLINT`	4	2558.08	1.56	7.57
KW13	`KWKKWVRAIKKLV`	4	1683.14	2.38	5.67
VK18	`VKRFRRWWKPWRKILHLV`	16	2505.07	6.39	5.98
RV15	`RVLWIKRWIKRFFRP`	8	2101.59	3.81	5.25
IR20	`IRFYWRRVQVWRGIWRRLVR`	32	2802.34	11.42	8.77
LW14	`LWRFKRWWWWKKIL`	16	2132.60	7.50	8.12
GI16	`GIKKFAKLLKYIAGKL`	16	1791.27	8.93	3.62
RR16	`RRWGKLIKKIAKKFGG`	8	1886.34	4.24	4.04
YK19	`YKWVANVAKKLIKLLKVLK`	32	2255.87	14.19	2.64
AK15	`AKKFKIWGAIKRLLA`	8	1743.19	4.59	4.56
KWI18	`KWKWPPRWPPWRPVWKVI`	32	2442.95	13.10	8.63

Table S3: Experimental and predicted MIC values of AMPs against *S. aureus* from AMP-Designer

Peptide	Sequence	Experiment MIC (μg/mL)	Mass (Da)	Experiment† MIC (μM)	Prediction MIC (μM)
RR13	`RRWFKIRMAAKLA`	64	1647.05	38.86	5.44
KW20	`KWRKVRAKFWRKWLAGLINT`	16	2558.08	6.25	4.68
VK18	`VKRFRRWWKPWRKILHLV`	16	2505.07	6.39	4.92
RK17	`RKKWWAYLLAKIAKKVK`	32	2130.67	15.02	7.49
GI16	`GIKKFAKLLKYIAGKL`	8	1791.27	4.47	6.59
YK19	`YKWVANVAKKLIKLLKVLK`	16	2255.87	7.09	5.33
AK15	`AKKFKIWGAIKRLLA`	8	1743.19	4.59	11.57
KWI18	`KWKWPPRWPPWRPVWKVI`	32	2442.95	13.10	9.29
LF19	`LFKVIFGIVKKKKLLPKFF`	8	2293.96	3.49	59.66
AV20	`AVWRWLWKGSLAKGIIKFLK`	8	2400.95	3.33	4.31

Table S4: Experimental and predicted MIC values of AMPs against *E. coli* from AMPd-Up

Peptide	Sequence	Experiment MIC (μg/mL)	Mass (Da)	Experiment[†] MIC (μM)	Prediction MIC (μM)
DeNo1002	NLLDTLKNLAKKLAKKLLKKLLKKL	3	2889.69	1.04	6.45
DeNo1003	NLLSTLLDAAKKAAKGAAKSAAKKLAKKLAKKL	3	3364.12	0.89	10.05
DeNo1004	HLLSGLLSAAKKAAKKAAKKALKKLLKKLLKKL	64	3553.55	18.01	10.72
DeNo1006	NLLDTLKKKAKKVAKKVLKKLLKKLLKKL	12	3373.39	3.56	15.51
DeNo1009	DLLKTLGKAAKKAAKTALKAALKGLLKKLAKKL	12	3446.35	3.48	8.23
DeNo1010	VLGGLLKKLLKKLLKKL	4	1905.54	2.10	12.67
DeNo1011	HLLSLLKKAAKKLLKKLLKKLAKKL	32	2868.76	11.15	18.25
DeNo1013	KIFGKILKKLLKKLLKKLLKKL	16	2635.54	6.07	22.50
DeNo1014	ALPSLLKKLAKKLAKKLLKKLLKKLLKKLLKKL	64	3794.06	16.87	13.29
DeNo1016	FLPIIAGLAAKFLPKIFCKITKKC	4	2664.41	1.50	14.53
DeNo1017	FLPIIAGLAAKLLPKLFCKITKKC	1.5	2630.39	0.57	13.59
DeNo1019	FLPKIAGKAAKKLPKIFCKITKKC	16	2675.43	5.98	8.54
DeNo1021	KLFGKLLKKLKKILKKIAKKIKKKL	8	2977.98	2.69	7.93
DeNo1023	DLLKTLKKIAKKLLKTLLKKLLKKLLKKL	128	3415.50	37.48	21.87
DeNo1027	RLPSIIPGIAGKLGGLLGGLLKGL	32	2313.87	13.83	13.81
DeNo1030	RLPRIFRGIRGKL	128	1581.95	80.91	19.72
DeNo1031	PLPPIIPGIAGKLLGGLLGLLKKL	12	2392.06	5.02	17.34
DeNo1040	MLPSILGALLKLL	96	1381.81	69.47	25.22
DeNo1045	FLPKIFKKIAKKL	96	1574.05	60.99	43.87
DeNo1046	VLGSLLKGLLKKL	24	1381.79	17.37	26.77
DeNo1048	LLPSLLKGLLKKL	128	1435.88	89.14	31.53
DeNo1049	ALLSLLKKLLKKL	6	1480.96	4.05	30.47
DeNo1051	ALPSLLKKLLKKL	24	1464.92	16.38	27.66
DeNo1057	ILGKLLKKLLKKL	12	1508.03	7.96	33.01
Ranateurin-4	FLPFIARLAAKVFPSIICSVTKKC	12	2653.30	4.52	12.87

Table S5: Experimental and predicted MIC values of AMPs against *S. aureus* from AMPd-Up

Peptide	Sequence	Experiment MIC (μg/mL)	Mass (Da)	Experiment[†] MIC (μM)	Prediction MIC (μM)
DeNo1001	DLLSGLGKAAKKVAKTVLKNLLKC	80	2512.11	31.85	8.32
DeNo1002	NLLDTLKNLAKKLAKKLLKKLLKKL	96	2889.69	33.22	7.86
DeNo1007	FLPSIIKGAAKKLPKIFCKILKKC	4	2688.47	1.49	4.30
DeNo1010	VLGGLLKKLLKKLLKKL	48	1905.54	25.19	4.67
DeNo1018	WLPKIAGKIAGKLLKKLLKKIKKK	8	2744.58	2.91	2.68
DeNo1021	KLFGKLLKKLKKILKKIAKKIKKKL	48	2977.98	16.12	9.68
DeNo1022	GLLSLLKKIGKKIGKLL	4	1822.37	2.19	10.15
DeNo1057	ILGKLLKKLLKKL	32	1508.03	21.22	2.87
Ranateurin-4	FLPFIARLAAKVFPSIICSVTKKC	3	2653.30	1.13	2.88
